# Curcumin and *Curcuma longa* L. extract ameliorate lipid accumulation through the regulation of the endoplasmic reticulum redox and ER stress

**DOI:** 10.1038/s41598-017-06872-y

**Published:** 2017-07-26

**Authors:** Hwa-Young Lee, Seung-Wook Kim, Geum-Hwa Lee, Min-Kyung Choi, Han-Wool Chung, Yong-Chul Lee, Hyung-Ryong Kim, Ho Jeong Kwon, Han-Jung Chae

**Affiliations:** 1 Department of Pharmacology and New Drug Development Institute, Chonbuk National University Medical School, Jeonju, Chonbuk 561-180 Republic of Korea; 2 Chemical Genomics Global Research Laboratory, Department of Biotechnology, College of Life Science and Biotechnology, Yonsei University, Seoul, 120-752 Republic of Korea; 3 Department of Internal Medicine, School of Medicine, Chonbuk National University, Jeonju, 560-182 Republic of Korea; 4 Daegu Gyeonbuk Institute of Science & Technology (DGIST) graduate school, Daegu Gyeonbuk Institute of Science & Technology (DGIST) graduate school, Daegu, Gyeonbuk South Korea

**Keywords:** Metabolic syndrome, Obesity

## Abstract

For this study, we examined the effects of curcumin against acute and chronic stress, paying specific attention to ROS. We also aimed to clarify the differences between acute and chronic stress conditions. We investigated the effects of curcumin against acute stress (once/1 day CCl_4_ treatment) and chronic-stress (every other day/4week CCl_4_ treatment). Compared with acute stress, in which the antioxidant system functioned properly and aspartate transaminase (AST) and ROS production increased, chronic stress increased AST, alanine aminotransferase (ALT), hepatic enzymes, and ROS more significantly, and the antioxidant system became impaired. We also found that ER-originated ROS accumulated in the chronic model, another difference between the two conditions. ER stress was induced consistently, and oxidative intra-ER protein folding status, representatively PDI, was impaired, especially in chronic stress. The PDI-associated client protein hepatic apoB accumulated with the PDI-binding status in chronic stress, and curcumin recovered the altered ER folding status, regulating ER stress and the resultant hepatic dyslipidemia. Throughout this study, curcumin and curcumin-rich *Curcuma longa* L. extract promoted recovery from CCl_4_-induced hepatic toxicity in both stress conditions. For both stress-associated hepatic dyslipidemia, curcumin and *Curcuma longa* L. extract might be recommendable to recover liver activity.

## Introduction

Humans can be transiently or chronically exposed to environmental contaminants and pollutants. People transiently exposed to toxic materials who get adequate rest might never develop detectable or diagnosable clinical symptoms. Although toxins from transient exposure are eliminated through liver metabolism, toxins from chronic exposure tend to accumulate in the body, leading to pathological phenomena, including hepatic dyslipidemia. Distinctive features of hepatotoxicity include hepatic dysfunction with an increase in aspartate aminotransferase (AST) and alanine aminotransferase (ALT) and hepatic dyslipidemia, in which pathological reactive oxygen species (ROS) accumulation and related signaling are the likely involved mechanisms.

ROS accumulation is controlled by stress intensity or duration. Under transient stress, ROS lead to adaption processes through the antioxidant system to promote recovery from the stress. Under severe or chronic stress, the ROS are amplified in subcellular organelles, disturbing protein folding and secretion^1, 2^. During intra-ER disulfide-bond formation, ROS disorganize the coupling of the protein disulfide isomerase (PDI) system^3, 4^. Redox imbalance caused by the disturbance of PDI coupling is perceived by the cell as ER stress^5^, which induces the release of glucose-regulated protein 78 (GRP78) from the ER and activation of three signaling cascades: the protein kinase RNA-like ER-associated kinase (PERK) pathway, the inositol-requiring protein 1 alpha (IRE1-α) pathway, and the activating transcription factor (ATF)-6 pathway^6^. Because ER dysfunction halts the maturation of ER-folded proteins, many chronic diseases are associated with ER stress^4, 7–10^. ROS and the related antioxidant system work well in transient toxicity; however, ROS accumulation and related ER stress can be induced in chronic toxicity. The status of ROS accumulation and the amplification into sub-organelle, ER, might therefore be a criterion to define stress intensity, which is a hypothesis in this study^11^.

Carbon tetrachloride (CCl_4_) is often used as a representative model for hepatotoxicity. In the ER, CCl_4_ is rapidly metabolized by mixed-function cytochrome P450 oxygenases, resulting in the generation of the toxic byproducts CCl_3_ **·** and CCl_3_OO**·** ^12, 13^, which subsequently cause hepatotoxicity. In addition, CCl_4_ decreases secretion of the triglycerides that compose very-low-density lipoproteins and increases hepatic triglycerides in rats^14^. Thus, we used CCl_4_ in this study to model both acute and chronic hepatotoxicity and lipid dyslipidemia.

Curcumin [1,7-bis(4-hydroxy-3-methoxyphenyl)-1,6-heptadiene-3,5-dione] is the main active component of turmeric (*Curcuma longa* L.), which is a member of the ginger family (Zingiberaceae). Curcumin is a nutraceutical with wide-ranging potential therapeutic actions, including antioxidant, anti-inflammatory, anti-infectious, anti-fibrotic, and anticancer, in both cell and whole-animal disease models^15, 16^. There is also evidence that curcumin treatment can protect against liver injury caused by various factors, including thioacetamide, iron overdose, cholestasis, and ethanol^17^. We studied the effects of both curcumin and curcumin-rich *Curcuma longa* L. extracts in CCl_4_ models to determine their therapeutic potential against hepatotoxicity and hepatic dyslipidemia and their mechanism of action, with a particular focus on ER redox imbalance.

## Results

### Curcumin and *Curcuma longa* L. extract protect rats against acute or chronic CCl_4_ toxicity

The main components of the *Curcuma longa L*. extract are: curcumin (2269.2 ± 12.3 mg/100 g), bisdemethoxycurcumin (BDMC; 1283.5 ± 8.5 mg/100 g), and demethoxycurcumin (DMC; 1284.6 ± 7.0 mg/100 g), as identified using high-performance liquid chromatography (HPLC) analysis^18, 19^ (Supplementary Fig. 1 and Supplementary Tables 1 and 2). To determine the effects of one of the active components against liver injury, we used 200 mg/kg of curcumin or 100, 200, or 300 mg/kg of *Curcuma longa* L. extract to treat to rats injected with CCl_4_ and olive oil-injected control rats. Each group contained 10 rats, and we used two separately designed models. For the acute model (0.1 mL/100 g, CCl_4_, one treatment), curcumin or *Curcuma longa* L. extract was given each day for 3 days before CCl_4_ treatment and on injection day. For the chronic model (0.1 mL/100 g, CCl_4_ every other day for 4 weeks), curcumin or *Curcuma longa* L. extract was given each day for 3 days before CCl_4_ treatment and every day during the 4-week stress period.

**Figure 1:**
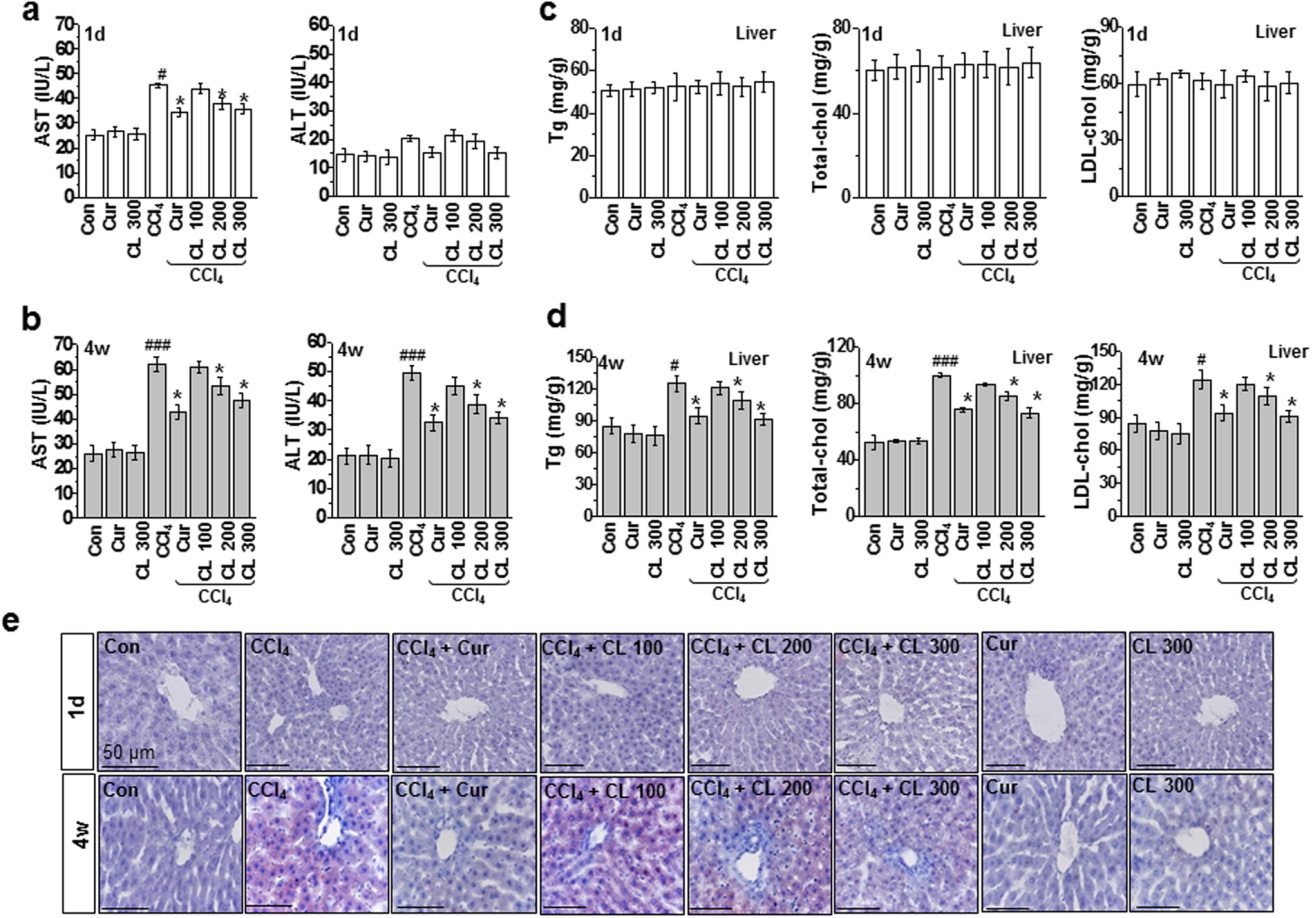
Curcumin and *Curcuma longa* L. extract regulate serum levels of AST and ALT and hepatic lipid accumulation in acute and chronic CCl_4_-models. Rats were intraperitoneally treated with CCl_4_ (0.1 mL/100 g, body weight) one time for (**a**) 1 day or (**b**) every other day for 4 weeks. Curcumin (200 mg/kg) or *Curcuma longa* L. extract (100, 200, or 300 mg/kg) was given each day for 3 days before CCl_4_ treatment and once daily after CCl_4_ treatment. Liver and blood samples were collected from all sacrificed animals. Six-h fasting serum levels of AST and ALT were determined. Six h fasting liver triglyceride, total cholesterol, and LDL-cholesterol levels were measured in the (**c**) 1 day and (**d**) 4 week CCl_4_-treated rats. (**e**) Representative images of liver sections from each group stained with hematoxylin-eosin and Oil-Red-O for lipid content. Scale bars = 50 µm. The experiments were repeated three times using tissues from at least three different rats. ^#^ *P* < 0.05, ^###^ *P* < 0.001 vs. the control group; ^*^ *P* < 0.01 vs. the CCl_4_ group (n = 10 rats per group). Cur: curcumin, CL: *Curcuma longa* L., AST: aspartate aminotransferase, ALT: alanine aminotransferase.

After 4 weeks, the average weight gain of rats in the control group (no CCl_4_ injection) was normal. In contrast, rats in the chronic model exhibited significantly increased weight gain compared with rats in the control group, but the administration of curcumin or *Curcuma longa* L. extract in the chronic model significantly inhibited weight gain (Supplementary Table 3). No significant differences were observed among any of the groups regarding daily food consumption. These findings suggest that both curcumin and *Curcuma longa* L. extract attenuate CCl_4_-induced weight gain. In the acute model, AST increased significantly, and ALT also increased, although not to a significant degree (Fig. 1a). Serum levels of ALT and AST were markedly elevated in the chronic model but correspondingly decreased with oral administration of curcumin and *Curcuma longa* L. extract (Fig. 1b). Staining with hematoxylin-eosin and Picro-Sirius revealed that the acute and chronic conditions caused no structural change or collagen deposition, suggesting neither hepatic necrosis nor fibrosis (Supplementary Fig. 2a and b).

### Curcumin and *Curcuma longa* L. extract regulate hepatic lipid metabolism in the chronic toxicity model

To investigate the effects of curcumin and *Curcuma longa* L. extract on hepatic lipid accumulation, we measured the levels of triglyceride (TG), total cholesterol, and LDL-cholesterol in liver tissues from the acute and chronic models. We found no difference in the lipid profiles between the control and CCl_4_-treated conditions in the acute model (Fig. 1c). However, liver TG, total cholesterol, and LDL-cholesterol levels all significantly increased in the chronic model, and curcumin or *Curcuma longa* L. extract correspondingly reduced the increased hepatic lipid profiles dose dependently (*Curcuma longa* L. extract) (Fig. 1d). Lipid droplets also accumulated less in the curcumin or *Curcuma longa* L. extract-treated conditions, compared with the untreated chronic condition (Fig. 1e). Consistent with these findings, serum lipid profiles were not affected by CCl_4_ in the acute model and were apparently regulated by curcumin or *Curcuma longa* L. extract in the chronic model (Supplementary Figs 3a–h and 4).

### Curcumin and *Curcuma longa* L. extract regulate hepatic ROS accumulation and enhance antioxidant system function in hepatotoxicity model

**Figure 2:**
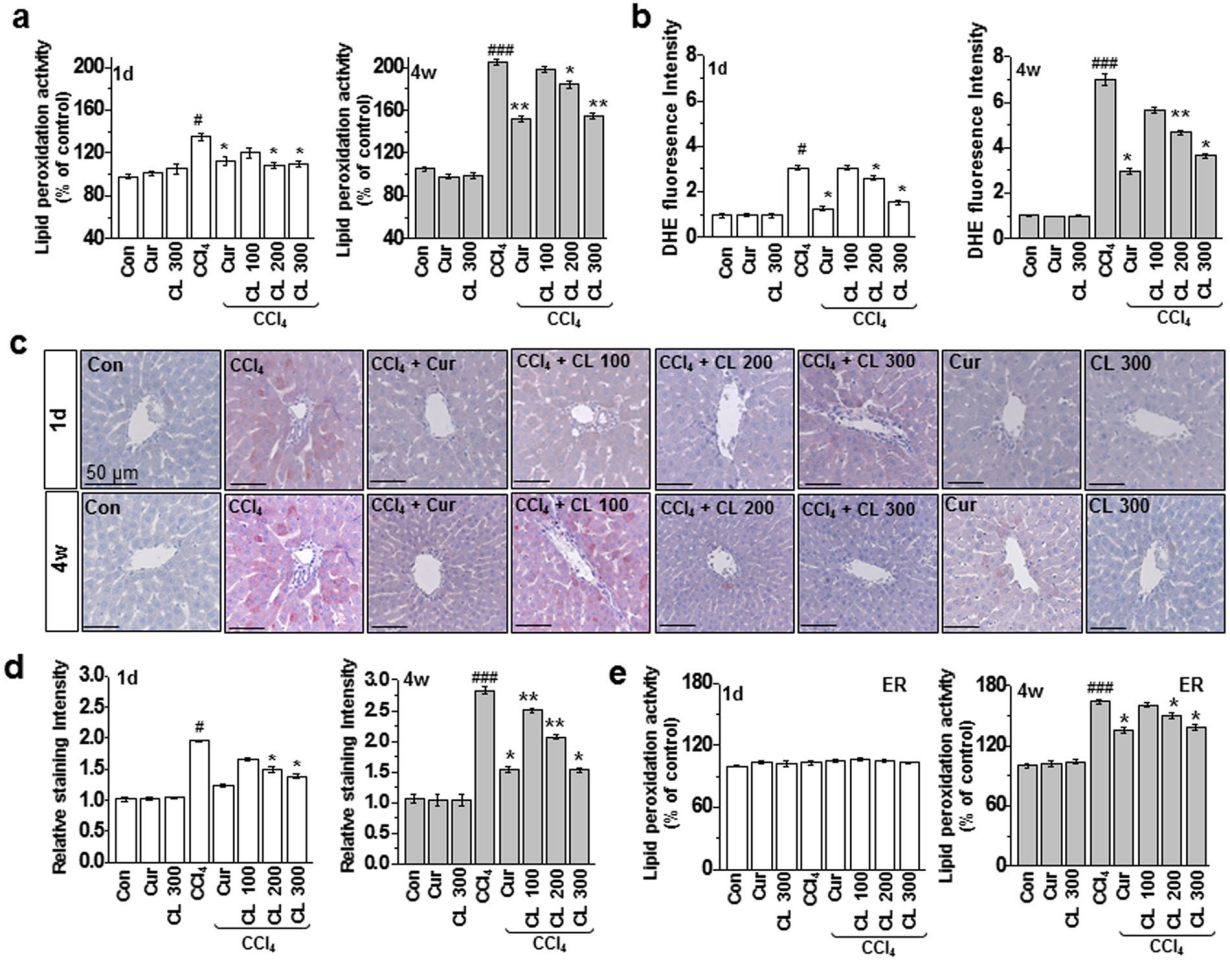
Curcumin and *Curcuma longa* L. extract regulate ROS accumulation in acute and chronic CCl_4_-models. Rats were intraperitoneally treated with CCl_4_ (0.1 mL/100 g body weight) one time for 1 day or every other day for 4 weeks. Curcumin (200 mg/kg) or *Curcuma longa* L. extract (100, 200, and 300 mg/kg) was given once daily. (**a**) Lipid peroxidation activity was measured in 1 day and 4 week CCl_4_-treated rats. (**b**) DHE staining in the liver was measured in 1 day and 4 week CCl_4_-treated rats. (**c**) Liver tissues from 1 day and 4 week CCl_4_-treated rats were stained with 4-HNE, and (**d**) the staining intensity of 4-HNE-positive cells was calculated. (**e**) Lipid peroxidation activity was measured in the ER fractions from the liver tissues of CCl_4_-treated rats. The experiments were repeated three times using tissues from at least three different rats. ^#^ *P* < 0.05, ^###^ *P* < 0.001 vs. the control group; ^*^ *P* < 0.01, ^**^ *P* < 0.05 vs. the CCl_4_ group (n = 10 rats per group). Cur: curcumin, CL: *Curcuma longa* L.

Due to the chemical metabolism of CCl_4_ into CCl_3_ **·**, ROS accumulation has been well studied in this toxicity model^14, 20^. However, acute and chronic exposure might affect liver detoxification and metabolic function differently due to the quantity of accumulated hepatic ROS. Therefore, we quantified lipid peroxidation levels in the acute and chronic conditions with and without the therapeutic candidates (curcumin or *Curcuma longa* L. extract). Compared with the acute condition, lipid peroxidation increased more in the chronic toxicity condition (Fig. 2a–d). Consistently, ROS production also increased more in the chronic condition than the acute condition (Fig. 2b). Interestingly, ER membrane lipid peroxidation was not observed in the acute toxicity condition. However, in the chronic model, ER lipid peroxidation was clearly observed, indicating that severe toxicity is linked to intra-ER redox imbalance/ROS accumulation, whereas mild toxicity-associated ROS are not amplified to subcellular organelles such as the ER (Fig. 2e). Curcumin and *Curcuma longa* L. extract suppressed the acute and chronic condition-associated ROS production/lipid peroxidation phenomenon.

Glutathione (GSH) has antioxidant activity due to the presence of a thiol group in its cysteine moiety, which acts as a reducing agent that can be reversibly oxidized and reduced^21^. GSH thus plays an important role in maintaining cellular antioxidant capacity. Chronic stress from CCl_4_ resulted in rapid depletion of hepatic GSH, and the GSH/glutathione disulfide (GSSG) ratio decreased significantly in the chronic model compared to the acute model (Supplementary Fig. 5a,b). Although in the endogenous antioxidant system, superoxide dismutase (SOD) and glutathione peroxidase (GPX) activity were maintained in acute stress, they were significantly disrupted in chronic stress (Supplementary Figs 5c,d and 6), but curcumin and *Curcuma longa* L. extract restored that decreased enzyme activity. Compared with the acute liver toxicity model, the intensity of ROS, the disturbed antioxidant system and the amplified redox imbalance into protein folding-organelle; ER are more significantly observed in the chronic toxicity model and curcumin and *Curcuma longa* L. extract regulate the redox-imbalance in the acute and chronic models, a main point in this study.

### Curcumin and *Curcuma longa* L. extract regulate hepatic apoB secretion and control the redox environment

**Figure 3:**
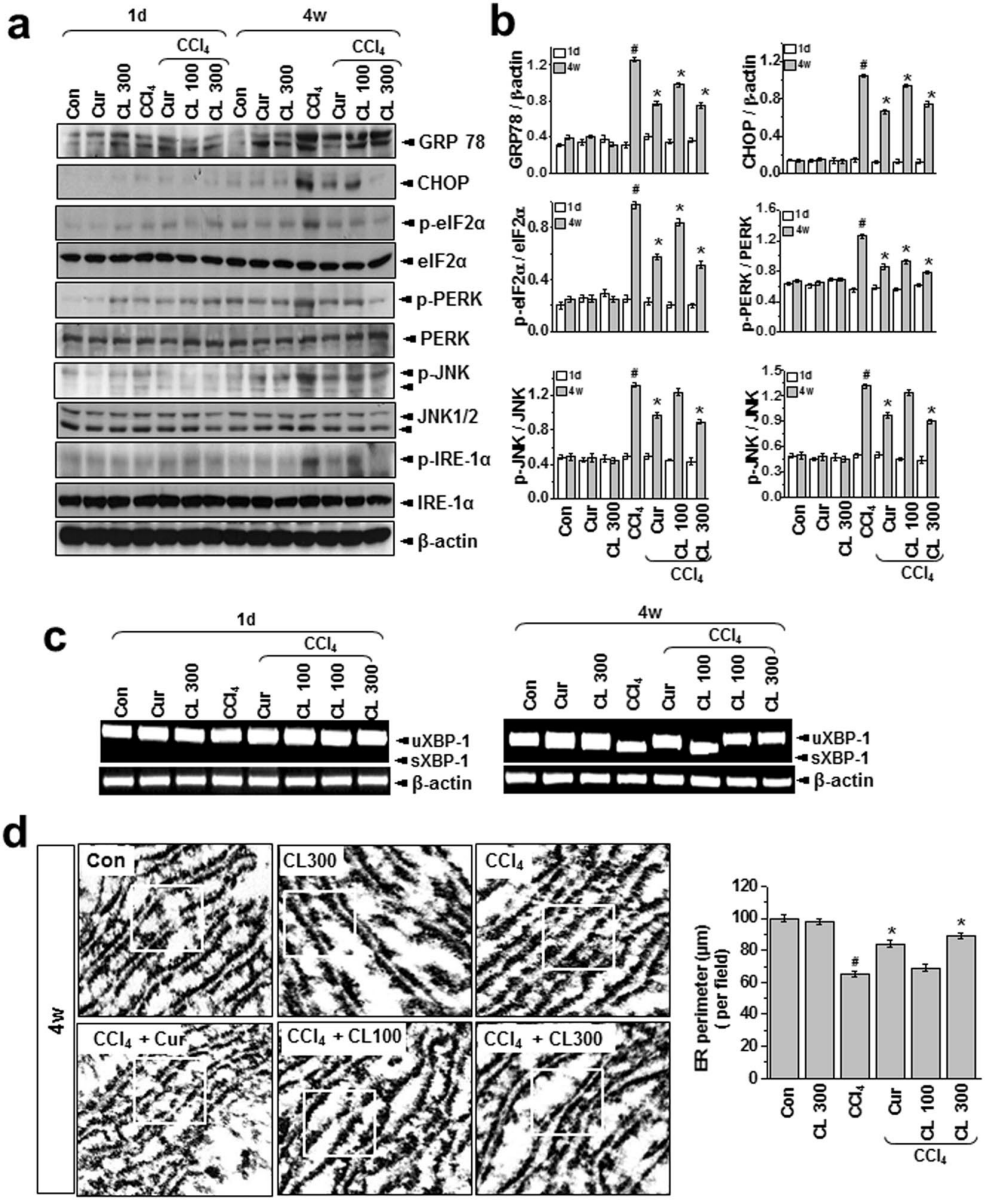
Curcumin and *Curcuma longa* L. extract inhibit CCl_4_-induced ER stress. (**a**) Rats were intraperitoneally treated with CCl_4_ (0.1 mL/100 g body weight) one time for 1 day or every other day for 4 weeks. Curcumin (200 mg/kg) or *Curcuma longa* L. extract (100 or 300 mg/kg) was given once daily. (**a**) Immunoblotting was performed using antibodies against anti-GRP78, CHOP, p-eIF2α, eIF2α, p-PERK, PERK, p-JNK, JNK, p-IRE-1α, IRE1-α, and β-actin. (**b**) The quantification analysis of antibody expression was performed using the indicated loading control. (**c**) sXBP-1 was measured with an RT-PCR assay as described in the text. (**d**) Representative electron microcopy images with clear ER morphology are shown. The experiments were repeated three times using tissues from at least three different rats. ^#^ *P* < 0.05 vs. the control group; ^*^ *P* < 0.01 vs. the CCl_4_ group (n = 10 rats per group). Cur: curcumin, CL: *Curcuma longa* L.

**Figure 4:**
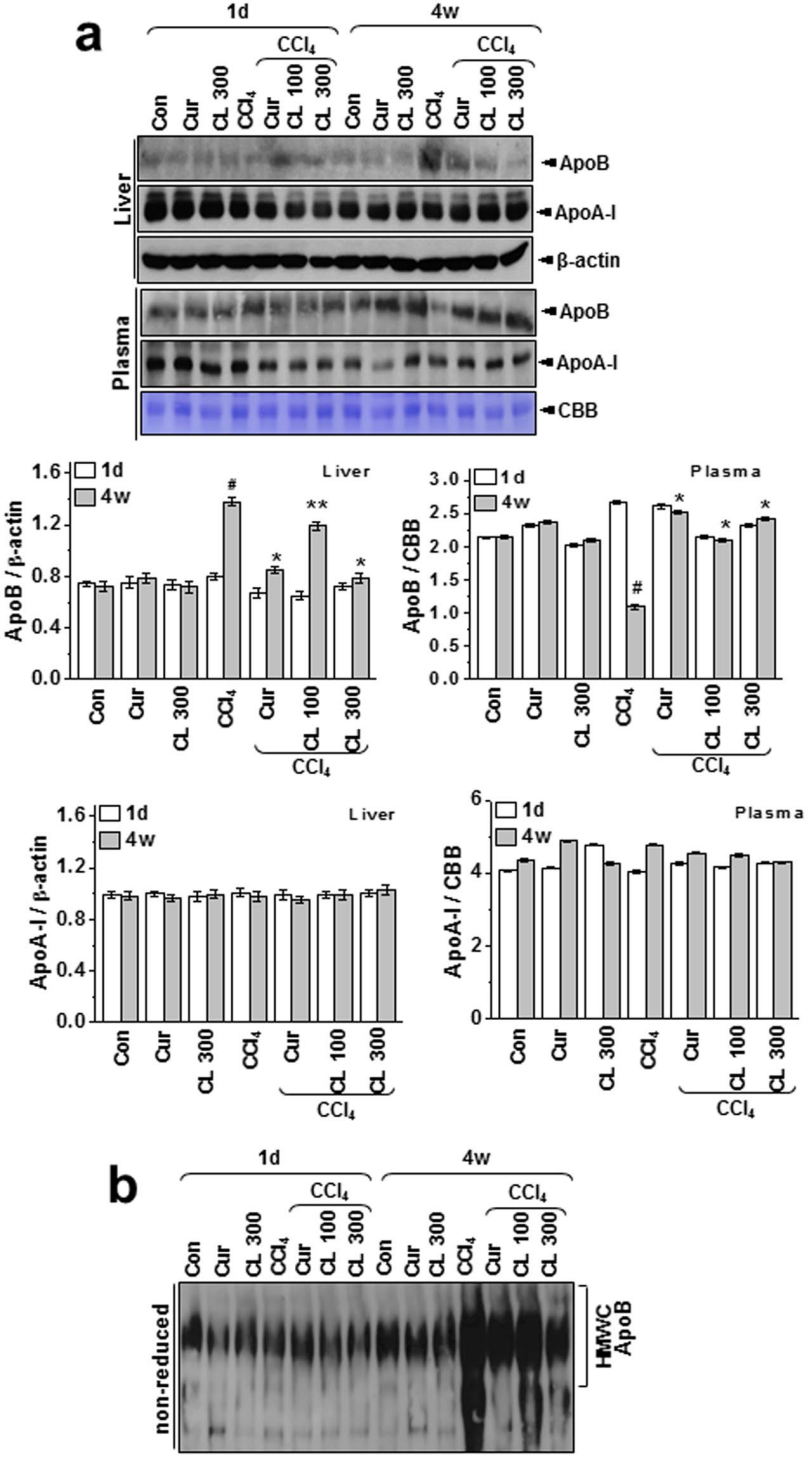
Curcumin and *Curcuma longa* L. extract regulate hepatic apoB secretion in acute and chronic CCl_4_-models. Rats were intraperitoneally treated with CCl_4_ (0.1 mL/100 g body weight) one time for 1 day or every other day for 4 weeks. Curcumin (200 mg/kg) or *Curcuma longa* extract (100 or 300 mg/kg) was given once daily. (**a**) Liver and plasma samples were subjected to immunoblot analysis with anti-apoB or anti-apoA-I antibody. CBB staining (albumin) was performed as a loading control for plasma. The quantification analysis of apoB and apoA-I expression was performed using the indicated loading control (β-actin for liver samples and albumin for plasma samples) (lower). (**b**) Non-reducing PAGE of liver lysates from the 1 day and 4 week CCl_4_-treated rats was used to confirm the presence of apoB in HMWC (n = 10 rats per group). The experiments were repeated three times using tissue from at least three different rats. ^#^ *P* < 0.05 vs. the control group; ^*^ *P* < 0.01, ^**^ *P* < 0.05 vs. the CCl_4_ group (n = 10 rats per group). Cur: curcumin, CL: *Curcuma longa* L, HMWC: high-molecular-weight complexes.

To investigate the difference in intra-ER lipid peroxidation status between the acute and chronic conditions and the regulatory effects of curcumin and *Curcuma longa* L. extract, we analyzed ER stress response in both toxicity conditions with and without curcumin and *Curcuma longa* L. The expression of ER stress marker proteins (GRP78, CHOP, p-eIF2α, p-PERK, p-IRE-1α, and spliced XBP-1) increased significantly in the chronic model but not in the acute model, and curcumin or *Curcuma longa* L. extract suppressed the ER stress response (Fig. 3a–c). Next, we used electron microscopy to investigate the effects of CCl_4_ treatment on liver morphology with and without curcumin or *Curcuma longa* L. extract. Those analyses revealed severely enlarged ER lumens in the chronic toxicity condition but not in the acute toxicity condition (Fig. 3d and Supplementary Fig. 7). As previous reports have indicated, cells under ER stress with compromised ER homeostasis often show ER enlargement^22–24^, suggesting that at least the chronic model causes ER stress. To explore the mechanism of CCl_4_-induced hepatic lipid-metabolism alteration, we analyzed the expression of representative apolipoproteins, apoA-I and apoB. In liver lysates from the chronic model, apoB, but not apoA-I, was highly induced after CCl_4_ injury, and curcumin and *Curcuma longa* L. extract reversed that hepatic apoB accumulation. Conversely, in plasma from the chronic model, the level of secreted apoB decreased after CCl_4_ injury, and that decrease reversed following curcumin or *Curcuma longa* L. extract treatment (Fig. 4a). The expression of apoB was unchanged in all test conditions in the acute model. In an apoB protein status assay using a non-native gel system, apoB aggregates were more evident in the chronic model than the acute model (Fig. 4b), indicating that curcumin and *Curcuma longa* L. extract attenuated the CCl_4_-induced hepatic apoB aggregation.

**Figure 5:**
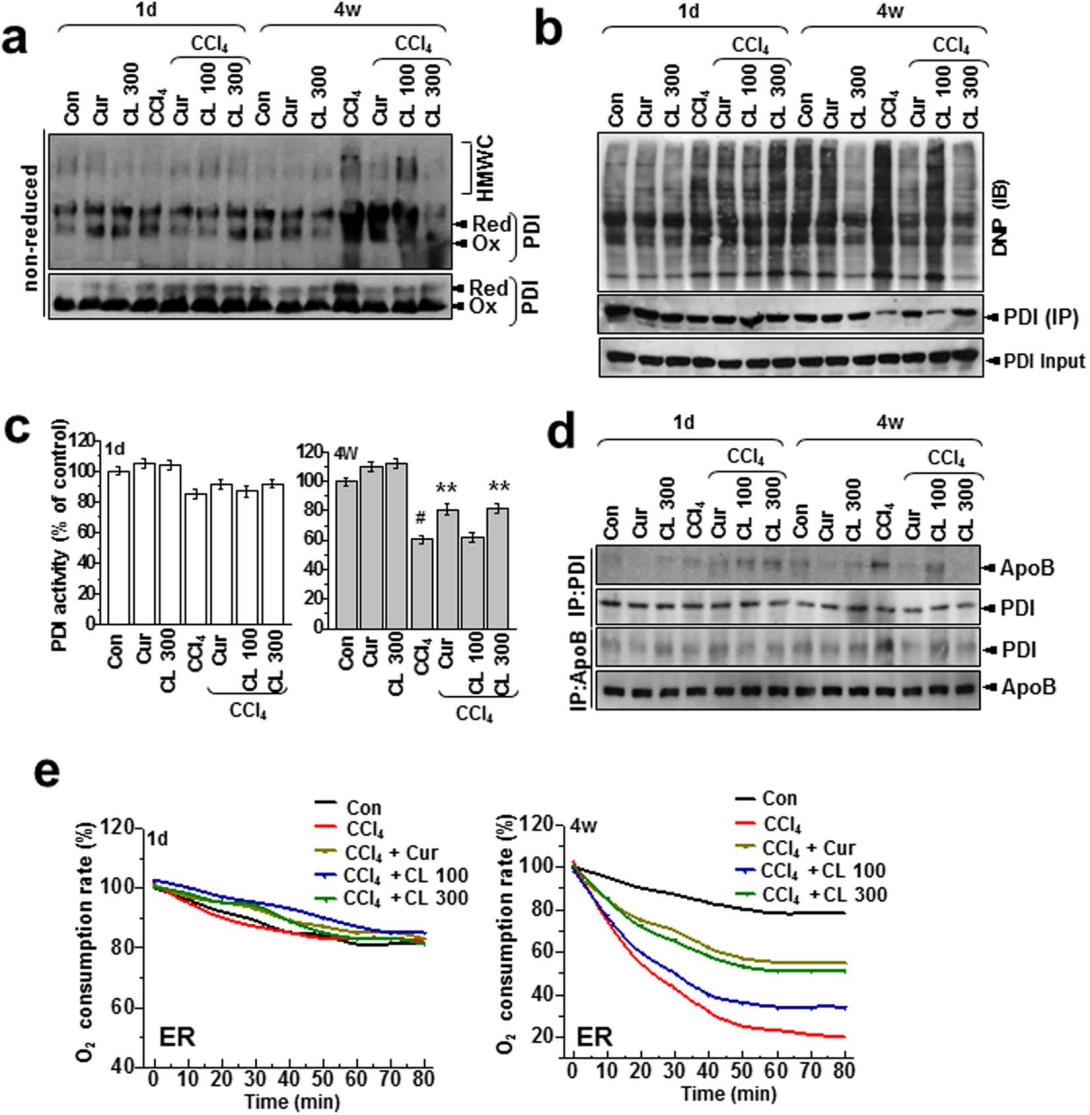
Curcumin and *Curcuma longa* L. extract regulate the formation of disulfide bonds during oxidative protein folding. (**a**) Liver lysates were analyzed for the presence of PDI in high-molecular-weight complexes using non-reducing gels. (**b**) Liver lysates were derivatized with DNPH, electrophoresed under non-reducing conditions, and immunoblotted with anti-DNP antibody. Independently, liver samples were immunoprecipitated and immunoblotted with anti-PDI antibody in reducing gel condition and immunoblotted with anti-PDI antibody. (**c**) PDI-reductase activity was measured in the same-treated liver samples. Results are presented as percentages of the PDI activity of control rats. (**d**) Liver lysates were immunoprecipitated with anti-PDI or anti-apoB antibody and immunoblotted with anti-apoB or anti-PDI antibody, respectively. (**e**) Oxygen-consumption kinetics were assayed using isolated ER fractions from 1 day and 4 week CCl_4_-treated rats. The experiments were carried out three times with similar activity profiles. The experiments were repeated three times using tissue from at least three different rats. CL: *Curcuma longa* L., PDI: protein disulfide isomerase, DNPH: 2,4-dinitrophenylhydrazine, DNP: dinitrophenol.

The ER quality-control machinery senses when a client protein achieves its native conformation and is released from the chaperone-PDI complex for export from the ER^25^. When the folding of client proteins (such as apoB) is compromised, the apoB that does not assume the mature conformation persists in multiprotein complexes called high-molecular-weight complexes (HMWC)^26^. These HMWC were elevated in liver samples from the chronic model but not in those from the acute model (Fig. 5a; PDI redox status more clearly shown under non-reducing conditions, bottom panel). HMWC formation in the chronic condition was attenuated by curcumin or *Curcuma longa* L. extract. Protein carbonylation, a type of protein oxidation, was also analyzed. In liver lysates from the chronic model, PDI was found to be excessively carbonylated after CCl_4_ injury, with most of the oxidized PDI corresponding to the fraction residing within HMWC (Fig. 5b). Also, the total amount of PDI decreased after CCl_4_ injury (Fig. 5b, lower), suggesting that the oxidized PDI residing within the HMWC-fraction is associated with client proteins (e.g., apoB) that failed to fold properly. Curcumin and *Curcuma longa* L. extract decreased the level of oxidized PDI in the HMWC fraction. Hepatic PDI activity was also significantly inhibited in the chronic model, and it was reversed by curcumin and *Curcuma longa* L. extract (Fig. 5c). Our analysis of the interaction between PDI and apoB showed that PDI was stably bound to apoB in liver samples from the chronic model and bound to a lesser extent in samples from the acute model (Fig. 5d), suggesting that apoB failed to fold properly and remained complexed with PDI, especially in the chronic condition. Curcumin or *Curcuma longa* L. extract effectively dissociated the interaction between PDI and apoB (Fig. 5d). For every disulfide introduced into a substrate protein, one molecule of hydrogen peroxide can theoretically be produced by the PDI and ER-oxidase1α (Ero1α) coupling system, providing a potential source of ROS and ER-generated oxidative stress^27^. To measure ER-originated ROS production, we used a fluorescence-based assay to monitor the consumption of oxygen. In samples from the chronic model, the rate of intra-ER oxygen consumption was faster than in samples from the acute model, indicating that the PDI-ERO1α coupling system is altered toward high O_2_ consumption and high production of ROS, especially in the chronic toxicity condition. The changed intra-ER O_2_ consumption recovered significantly with treatment by curcumin or *Curcuma longa* L. extract (Fig. 5e).

### Curcumin and *Curcuma longa* L. extract regulate the redox environment and ER stress in hepatocytes

To examine the physiological relevance of *in vivo* observations, we examined the effects of curcumin and *Curcuma longa* L. extract on the hepatic redox environment in CCl_4_-treated primary hepatocytes. When primary hepatocytes were treated with CCl_4_ for 1, 2, 4, 6, 12, and 24 h, the levels of whole cell and ER membrane lipid peroxidation changed as shown in Supplementary Fig. 8. Lipid peroxidation in primary hepatocytes increased significantly starting from 6 h-treatment whereas ER specific lipid peroxidation was not observed at the 6 h-treatment but observed after 12 h-treatment condition (Supplementary Fig. 8a,b). Based the ER-associated ROS accumulation, a suggestive criteria about chronic stress in this study was only observed from 12 h-treatment, we selected the 6 h-treatment condition as an acute model and 12 h-treatment as a chronic model.

**Figure 6:**
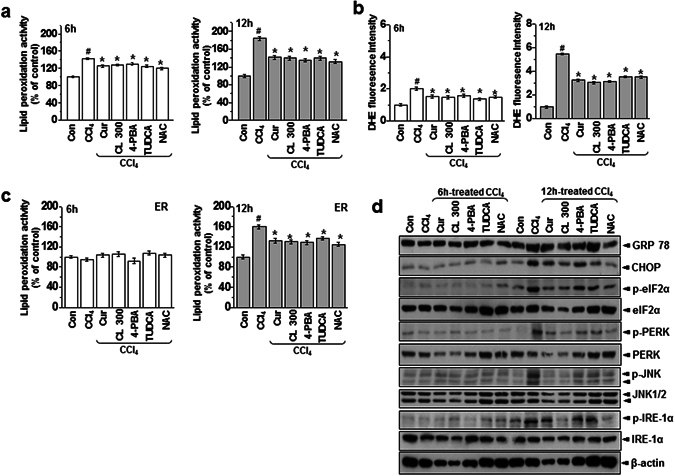
Curcumin and *Curcuma longa* L. extract inhibit CCl_4_-induced ROS accumulation and ER stress. Mouse primary hepatocytes were exposed to 0.5% CCl_4_ with or without 2 µM curcumin, 25 µM CL, 5 mM 4-PBA, 500 ug/mL TUDCA, or 2 mM NAC for 6 h or 12 h. (**a**) Lipid peroxidation activity was measured in 6 h or 12 h CCl_4_-treated hepatocytes. (**b**) DHE staining in the liver was measured in 6 h or 12 h CCl_4_-treated hepatocytes. (**c**) Lipid peroxidation activity was measured in the ER fractions of hepatocytes from 6 h or 12 h CCl_4_-treated hepatocytes. (**d**) Immunoblotting was performed using antibodies against anti-GRP78, CHOP, p-eIF2α, eIF2α, p-PERK, PERK, p-JNK, JNK, p-IRE-1α, IRE1-α, and β-actin. The experiments were repeated three times using at least three different samples. ^#^ *P* < 0.05 vs. the control group; ^*^ *P* < 0.05 vs. the CCl_4_ group. Cur: curcumin, CL: *Curcuma longa* L, 4-PBA: 4-Phenylbutyric acid, TUDCA: Tauroursodeoxycholic acid, NAC: N-Acetyl-L-cysteine.

**Figure 7:**
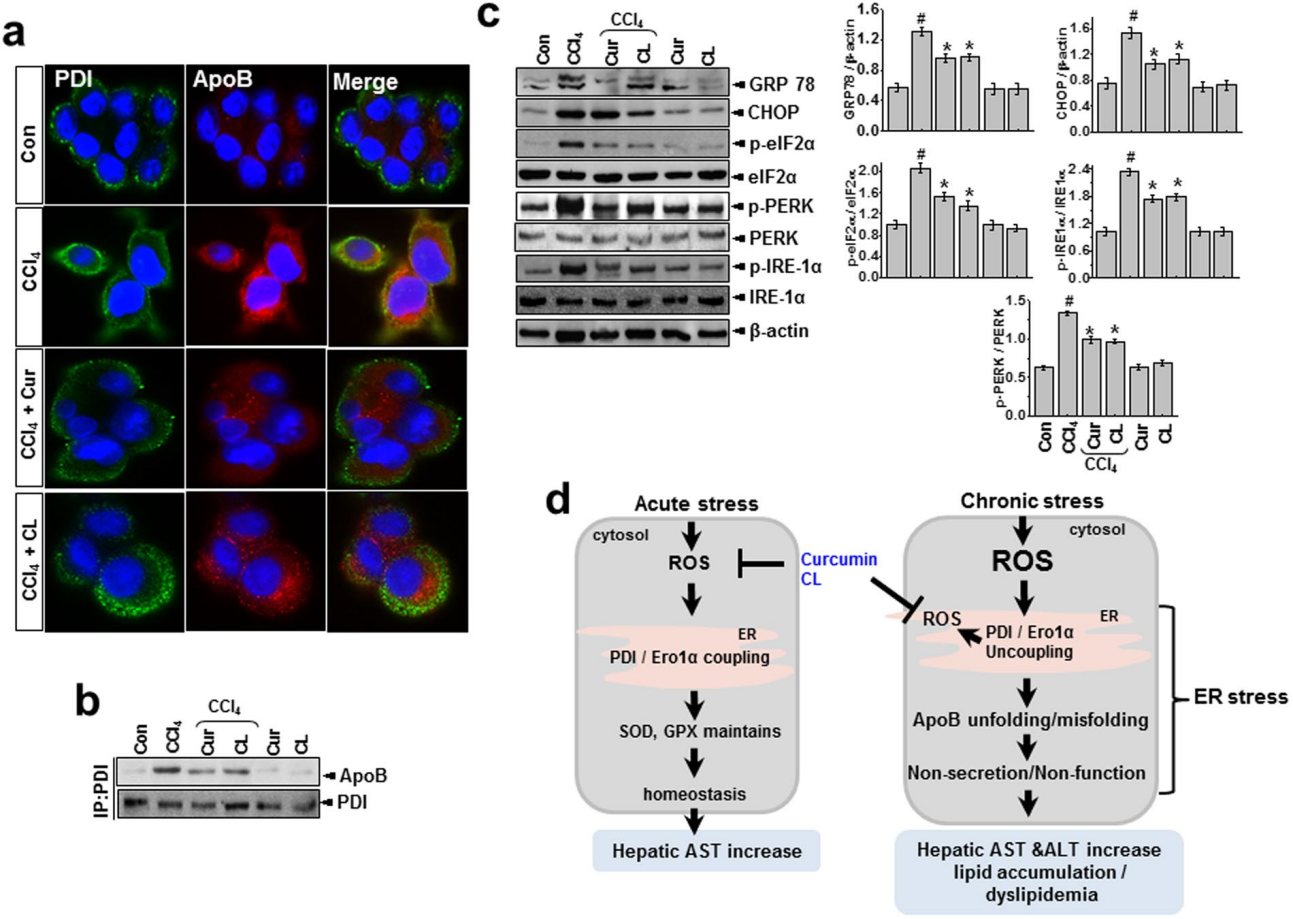
Curcumin and *Curcuma longa* L. extract regulate interaction between PDI and apoB, inhibiting ER stress in CCl_4_-treated primary hepatocytes. Mouse primary hepatocytes were exposed to 0.5% CCl_4_ with or without 2 µM curcumin or 25 µM CL for 12 h. Hepatocytes were assessed by confocal microscopy (scale bar, 10 µm). Images show the localization of PDI (green) and apoB (red) in the ER. Cell nuclei were stained with DAPI (blue). (**b**) Protein extracts from hepatocytes were immunoprecipitated with anti-PDI or apoB antibody and immunoblotted with anti-apoB or anti-PDI antibody, respectively. (**c**) Immunoblotting was performed using antibodies against anti-GRP78, CHOP, p-eIF2α, eIF2α, p-IRE-1α, IRE1-α, and β-actin. (**d**) Schematic representation of signaling in acute or chronic CCl_4_-induced dyslipidemia. The experiments were repeated three times using at least three different samples. ^#^ *P* < 0.05 vs. the control group; ^*^ *P* < 0.05 vs. the CCl_4_ group. Cur: curcumin, CL: *Curcuma longa* L., PDI: protein disulfide isomerase.

Compared with the acute condition, lipid peroxidation in primary hepatocytes increased more in the chronic toxicity condition (Fig. 6a). ROS production also increased more in the chronic condition than the acute condition (Fig. 6b). Similar to the *in vivo* models, we did not observe ER membrane lipid peroxidation in the acute toxicity condition. In the chronic model, however, ER lipid peroxidation was clear, indicating that severe toxicity is linked to intra-ER redox imbalance/ROS accumulation, whereas ROS associated with mild toxicity are not amplified to subcellular organelles such as the ER (Fig. 6c). Treatment with curcumin or *Curcuma longa* L. extract suppressed both acute and chronic condition–associated ROS production and lipid peroxidation. To investigate the difference in intra-ER lipid peroxidation status between acute and chronic conditions and the regulatory effects of curcumin and *Curcuma longa* L. extract, we analyzed ER stress response in acute and chronic toxicity conditions with and without curcumin and *Curcuma longa* L. extract. The expression of the ER stress marker proteins (GRP78, CHOP, p-eIF2α, p-PERK, and p-IRE-1α) increased significantly in the primary hepatocytes in the chronic model but not in the acute model, and curcumin or *Curcuma longa* L. extract suppressed that ER stress response (Fig. 6d). To find a specific molecular mechanism underlying the relationship between PDI and apoB, we first assessed the subcellular localization of apoB following CCl_4_ treatment. Using microscopy, we used an anti-PDI antibody that recognizes ER chaperones to visualize the ER. After CCl_4_ treatment of primary hepatocytes, the induced apoB protein was placed in punctae along the tubular ER network, co-localizing with PDI proteins. Treatment with curcumin and *Curcuma longa* L. extract clearly reversed the increase in the fluorescence/colocalized pattern in CCl_4_-treated cells (Fig. 7a). We also examined the interaction between PDI and apoB in hepatocytes. PDI was stably bound to apoB in CCl_4_-treated primary hepatocytes and to a lesser extent in hepatocytes treated with curcumin or *Curcuma longa* L. extract (Fig. 7b). The levels of the ER-stress proteins (GRP78, CHOP, p-eIF2α, p-PERK, and p-IRE-1α) in primary hepatocytes increased significantly after CCl_4_-treatment, and curcumin or *Curcuma longa* L. extract reduced this over-expression (Fig. 7c), indicating that curcumin and *Curcuma longa* L. extract protect against CCl_4_-induced intra-ER apoB accumulation and ER stress in primary hepatocytes. In summary, chronic stress–induced ROS become intra-ER ROS that cause PDI/ERO-1α uncoupling, which results in ER stress and apoB aggregation-associated hepatic dyslipidemia. On the other hand, acute stress–induced ROS enter homeostasis physiology by enhancing ER folding and controlling ER stress through PDI activity, in addition to regulating the antioxidant system (Fig. 7d).

## Discussion

In this study, we examined the difference between acute and chronic stress in terms of ROS, along with the effects of curcumin and *Curcuma longa* L. extract against acute and chronic stress. Compared with acute stress, more ROS accumulated during chronic stress. In the acute model, the endogenous antioxidant system, i.e., SOD and GPX activity, functioned normally, but not in chronic model. In other words, the adaptation system to protect against ROS functions in acute stress but not during chronic stress. This study also showed that intra-ER ROS specifically accumulated and oxidative intra-ER protein folding status became impaired in the chronic model, which is another way to explain the difference between the two conditions. Consistently, apoB, a representative apolipoprotein, was ineffectively folded, and as a result, hepatic lipid was accumulated during chronic stress. Curcumin and *Curcuma longa* L. extract inhibit hepatic ROS accumulation in both acute and chronic stress and correct the altered ER folding status and resulting hepatic dyslipidemia during chronic stress.

### Hepatic lipid dysmetabolism and related oxidative folding disturbances are induced during chronic toxicity

Humans are frequently exposed to acute or chronic stress from diverse sources, including the environment. This study uses a hepatotoxicity model to show that chronic stress, but not acute stress, is linked to hepatic lipid accumulation through the intrahepatic aggregation of the apolipoprotein apoB. The hepatic enzyme AST (but not ALT) increased after acute stress induced by CCl_4_ exposure. However, in the 4-week chronic stress model, both ALT and AST increased significantly more than in the acute model (Fig. 1). In the chronic condition, hepatic TG, total cholesterol, and LDL-cholesterol also increased significantly, whereas they were unaffected in the acute condition. Therefore, the chronic condition, but not the acute condition, is closely related to pathology. Consistently, apoB, a key apolipoprotein for the transfer of TG and cholesterol, was accumulated in liver lysates from the chronic condition (Fig. 3), suggesting that apoB becomes a client target protein for PDI, an ER chaperone, which results in ER stress during chronic stress (Fig. 5). More specifically, we also verified that carbonylated proteins were highly associated with PDI, especially in the chronic toxicity model (Fig. 4b). PDI’s presence within the HMWC fraction suggests that most oxidized PDI is associated with client proteins. If PDI works effectively, it releases its folded client proteins to the appropriate subcellular organelles and does not appear within the HMWC fraction^28–30^. In the chronic toxicity model, abnormal oxidation of PDI by CCl_4_-induced ROS could be a primary cause of the ER stress response. In the chronic hepatotoxicity condition, the ratio of GSH to GSSG (and the linked GPX and SOD) decreased significantly compared to the acute condition (Supplementary Figs 5 and 6), which reflects the ROS levels in each toxicity model (Fig. 2). High or continuous production of ROS can alter the redox status, oxidative stress in the ER lumen, and ER stress^3, 31–33^. In an endogenously maintained antioxidant system, ROS is not linked to disturbance of lipid homeostasis, but above some specific threshold of ROS, the amplified ROS in subcellular organelles are linked to ER stress, and key proteins are disturbed at the folding step, ultimately leading to hepatic dyslipidemia. Based upon the ER membrane lipid peroxidation analysis in CCl_4_-treated cells (Supplementary Fig. 8b), we consider the 12 h-treatment condition as a chronic stress in CCl_4_-exposed hepatocyte toxicity study. The stress condition also induces ER stress response and its related alteration of the secretion of the PDI-client protein, ApoB (Fig. 7a,c), which is well correlated with the suggested criteria between acute and chronic stress “Whether ER stress occurs or not”. Consistently, ER redox imbalance and ER membrane lipid peroxidation are clearly detected in the mentioned relatively chronic condition (Fig. 6a–c), suggesting that intra-ER redox imbalance and ER stress might to be a criteria to divide acute and chronic stress *in vitro* and *in vivo*.

### Curcumin and *Curcuma longa* L. extract regulate hepatic dyslipidemia and its related oxidative folding disturbances under chronic toxicity conditions

In this study, curcumin and *Curcuma longa* L. extract protected against acute and chronic stress by maintaining the redox balance through the antioxidant system and ER redox machinery. In antioxidant-related studies, curcumin decreased lipid storage in the hepatocytes of high-fat diet rodents^34^, and curcumin played a principal role in cellular redox control^35^. After induction by cholesterol, administration of curcumin reduced the concentration of lipid- and thiobarbituric acid–reactive substances in the liver and plasma^36^ through enhanced liver GSH-Px activity, suggesting that *Curcuma longa* L. extract and curcumin have diverse anti-oxidant activities that control ROS-associated liver toxicity. Studies about absorption, distribution, metabolism and excretion of curcumin have revealed poor absorption and rapid metabolism of curcumin that severely curtails its bioavailability^37^. We thus selected a high dose of curcumin, 200 mg/kg, during our experimental design. Other studies have used a similar dose of curcumin^38–42^. In this study, we expected the orally administered curcumin to be metabolized to a greater degree in the CCl_4_-exposed condition.

Because hepatocytes have a well-developed ER structure, ER stress is thought to be involved in liver-related diseases^41^. Curcumin has an inhibitory effect on ER stress^42^ in its role as an antioxidant, so we strongly suggest that curcumin and *Curcuma longa* L. extract effectively cover the ER folding requirement, showing an enhanced PDI-linked redox capability (Figs 4, 5 and 7) and also regulate ER stress signaling through GRP78, CHOP, p-eIF2α, p-PERK, p-IRE-1α, and sXBP-1 under CCl_4_ conditions (Fig. 3a). Taken together, our data suggest that the regulation of ER stress is the main mechanism by which curcumin and *Curcuma longa* L. extract counteract lipid dysmetabolism.

In summary, we found that curcumin and *Curcuma longa* L. extract regulate ROS through a counterbalance of antioxidants in acute stress and by controlling amplified ROS signaling and the subsequent ER stress in chronic hepatotoxicity. Our findings provide molecular evidence supporting curcumin and *Curcuma longa* L. extract as possible therapeutic agents for the management of hepatic dyslipidemia.

## Materials and Methods

### Materials

CCl_4_ and curcumin were purchased from Sigma-Aldrich (St. Louis, MO, USA). Malondialdehyde and GSH detection kits were obtained from BioVision (Mountain View, CA, USA). AST, ALT, total cholesterol, TG, HDL-cholesterol, and LDL-cholesterol detection kits were obtained from Asan Pharmaceutical Company (Seoul, Korea). Olive oil was purchased from CJ CHEIL HEDANG (Seoul, Korea). The purity of the olive oil was 100%, and it contained monounsaturated fatty acid (70% oleic acid and 3.5% palmitoleic acid), polyunsaturated fatty acid (15% linoleic acid and 0.5% α-linolenic acid), and saturated fats (13% palmitic acid and 1.5% stearic acid). There are 5 mg of polyphenols in every 10 grams of olive oil, and 1.6 mg of vitamin E is included per tablespoon of the olive oil.

### Preparation of *Curcuma longa* L. extract

*Curcuma longa* L. rhizomes were purchased from India (Rajapuri). The rhizomes were cleaned, dried, ground, and weighed, then homogenized in 50% ethanol for 3 days at 25 °C with occasional shaking and stirring. The mixture was filtered, and the resulting liquid was concentrated under reduced pressure at 45 °C in a rotary evaporator to yield a gummy, dark-yellow extract (9.8%, w/w). The concentrated extract was then kept in an incubator at 45 °C for 3 days to evaporate any ethanol residue, yielding the crude rhizome extract.

### Animal experiments

One hundred sixty male Sprague-Dawley rats weighing 250–270 g were obtained from Central Laboratory Animal, Inc. (Seoul, Korea) and randomly assigned into groups. The experimental animals were given free access to standard diet and water in rooms maintained at 25 °C on 12 h light/dark cycles. Rats were injected intraperitoneally (i.p.) with a mixture of CCl_4_ (0.1 mL/100 g) and olive oil [1:1(v/v)] one time for 1 day or every other day for 4 weeks. *Curcuma longa* L. extract was orally administered at doses of 100, 200, or 300 mg/kg body weight. Curcumin was given once daily at a dose of 200 mg/kg. The control animals were handled similarly, including i.p. injection with the same volume of olive oil, and oral administration of the same volume of phosphate-buffered saline (PBS). After the last CCl_4_ injection, the rats were anesthetized with diethyl ether (Sigma) and sacrificed. All animal procedures in this study were performed in accordance with the regulations described in the Care and Use of Laboratory Animals guide of Chonbuk National University. All procedures were also approved by the Institutional Animal Care and Use Committee of Chonbuk National University (IACUC protocol, CBNU 2017-0012).

### Blood and liver sample collection

Rats were sacrificed after a 16 h fast. Blood samples were collected, and serum was used for biochemical assays. Liver tissues were dissected quickly on ice. A portion of each liver was immediately fixed for histological analysis, and the remaining portion was stored in liquid nitrogen for biochemical and molecular analysis.

### Histological analysis of liver

Immediately after removal, rat liver portions were fixed for 1 day at room temperature in Carnoy’s fixative (ethanol:chloroform:acetic acid, 6:3:1), then preserved in 70% ethanol. Liver specimens were dehydrated with a graded series of alcohol preparations and embedded in paraffin. Specimens were cut in 7 μm sections using a rotary microtome and mounted on 3-aminopropyltriethoxysilane-coated glass slides. Each section was de-paraffinized in xylene, rehydrated in decreasing concentrations of alcohol in water, and stained with hematoxylin-eosin reagent (Sigma). The slide was then mounted with neutral balsam.

### Serum and liver tissue analyses

Liver lipids were extracted according to the method of Bligh and Dyer^43^, in which the chloroform layer was used to determine TG, total cholesterol, LDL-cholesterol, and HDL-cholesterol levels. Serum and hepatic TG, total cholesterol, LDL-cholesterol, and HDL-cholesterol levels and serum AST and ALT concentrations were each determined using the corresponding biochemical assay kits (Asan Pharmaceutical Company).

### Hepatocyte culture

Hepatocytes were isolated from the livers of mice using a modification of the collagenase method^44^ and seeded at a density of 0.5 × 10^6^ cells per 35 mm.

### Immunoblotting

Liver tissue homogenates were prepared by homogenizing the tissue in RIPA buffer (50 mM Tris, pH 8.0; 150 mM NaCl; 2 mM EDTA; 1% Nonidet P-40; 0.1% SDS) supplemented with a protease inhibitor cocktail tablet (Roche, Indianapolis, IN, USA) and phosphatase inhibitory cocktails 2 and 3 (Sigma-Aldrich). Lysates were cleared by centrifugation and analyzed by gel electrophoresis. Protein concentration was determined via the Bradford protein assay (Bio-Rad, Hercules, CA, USA) using bovine serum albumin (BSA) as a standard, and verified by Coomassie blue gel staining. Lysates (40 μg) were resolved by SDS-PAGE (Bio-Rad) and then transferred to nitrocellulose membranes. Membranes were blocked for 1 h with 5% skim milk in Tris-buffered saline (0.137 M NaCl, 0.025 M Tris, pH 7.4) containing 0.1% Tween-20. Antibodies were diluted according to the manufacturers’ recommended protocols. After incubation with an enhanced chemiluminescence (ECL) reagent (SuperDetect^TM^ ECL Western Blotting Detection Reagent, DaeMyung Science Co., Ltd, Seoul, Korea), the membranes were exposed to film (Amersham Hyperfilm TM ECL, GE Healthcare Limited, Buckinghamshire, UK) to detect protein signals.

### RNA isolation and reverse transcription polymerase chain reaction

Total RNA was isolated from liver using Trizol reagent (Invitrogen) according to the manufacturer’s manual. The sequences of the primers used for RT-PCR were as follows: total spliced XBP1 transcripts were quantified using the forward primer 5′-ACGAGAGAAAACTCATGGC-3′, the reverse primer 5′-ACAGGGTCCAACTTGTCCAG-3′, rat β-actin using forward primer 5′-ACCACCATGTACCCAGGCATT-3′, and the reverse primer 5′-CCACACAGAGTACTTGCGCTCA-3′. Samples were loaded onto an optical reaction plate, and reactions were run on a 2% agarose gel and viewed by UV illumination.

### Immunoprecipitation

For immunoprecipitation assays, mouse liver lysates were prepared in 50 mM Tris-HCl, pH 8.0, containing 150 mM NaCl, 0.015% phenylmethylsulfonyl fluoride, 1 mM dithiothreitol, 1 mM EDTA, 1% sodium deoxycholate, 1% Triton X-100, and 1% SDS. Liver lysates were incubated at 70 °C for 15 minutes to ensure complete cell lysis and were then diluted with lysis buffer to achieve an SDS concentration of 0.1% (w/v). Lysates were incubated with antibodies (1:100 dilution) for 1 h and then with 20 μL of protein A/G-Sepharose (10% solution) for an additional 2 h. Immunocomplexes were collected by centrifugation, washed three times with PBS, and eluted in 50 μL of sample buffer. Immunocomplexes were then separated using SDS-PAGE and visualized via exposure to a phosphoimaging screen.

### PDI redox state and high-molecular-weight protein complex formation assay

Procedures were performed as described previously^45^. Briefly, liver tissue was washed twice with ice-cold PBS supplemented with 20 mM N-ethylmaleimide (NEM) (to protect the reduced disulfide bonds from further oxidation during lysis), lysed in lysis buffer (20 mM Tris, pH 7.4; 150 mM NaCl; 0.5% Triton X-100) for 30 min on ice, and cleared by centrifugation. The protein concentration was determined using the Bradford protein assay, with BSA as a standard. Then, 50 μg of total protein was separated via native polyacrylamide gel electrophoresis. To distinguish between redox forms of PDI (no SDS, not boiled), 12% polyacrylamide non-reducing gels were electrophoresed using 50 V at 4 °C, and 8% polyacrylamide gels were used to detect complexes between PDI and its client proteins.

### PDI carbonylation assay

Proteins (100 μg) from liver tissue homogenates were derivatized for 5 minutes using the OxyBlot kit (Millipore, Billerica, MA, USA). The reaction was stopped with the addition of a neutralization solution, per the manufacturer’s instructions. To remove 2,4-dinitrophenylhydrazine (DNPH), proteins were pelleted by ultracentrifugation (Beckman TL-100 ultracentrifuge, TLA100.2 rotor, Beckman Coulter, Fullerton, CA, USA) at 100,000 × *g* for 1 h at 4 °C. Protein pellets were washed with immunoprecipitation (IP) lysis buffer and resuspended in 500 μL of IP lysis buffer. PDI was immunoprecipitated as described above, resolved in non-reducing gels, Western blotted, and probed with anti-DNP rabbit antibodies, per the OxyBlot kit instructions.

### PDI activity assay

A fluorescence-based insulin reduction assay (ProteoStat PDI Assay Kit, Enzo Life Sciences, Farmingdale, NY, USA) was used to measure cell surface PDI activity^43^. Tissues were harvested, resuspended in a 50 mM Tris-HCl, pH 7.5 buffer containing 25 mM KCl and 5 mM MgCl_2_ and then sonicated. After protein quantification, 500 μg of tissue lysates were assayed according to the manufacturer’s instructions. The fluorescence was then measured in a microplate reader using an excitation setting of 500 nm and an emission filter of 603 nm.

### Oxygen electrode assays with PDI

The oxygen consumption rate was detected using the cell-based Oxygen Consumption Rate Assay Kit (Cayman Chemical Company, Ann Arbor, MI, USA) according to the manufacturer’s protocol.

### Antioxidant enzymes

The activities of SOD and GPX were analyzed using assay kits from Cayman according to the manufacturer’s instructions (Cat. 706002 and 703102, Cayman, Ann Arbor, MI, USA).

### GSH/GSSG analysis

The levels of serum reduced and oxidized glutathione (GSH and GSSG) were measured using a kit from BioVision (Cat. K264, BioVision, Inc, CA, USA) according to the manufacturer’s protocol.

### ER fractionation

The microsomal fraction was obtained as previously described^46^. Briefly, liver tissues were resuspended in buffer A (250 mM sucrose, 20 mM HEPES, pH 7.5, 10 mM KCl, 1.5 mM MgCl_2_, 1 mM EDTA, 1 mM EGTA) and protease inhibitor complex (Roche Diagnostics, Mannheim, Germany) on ice for 30 min. The lysates were then homogenized and centrifuged at 750 × g for 10 min at 4 °C. The supernatant from the homogenized lysates was then centrifuged at 100,000 × g for 1 h at 4 °C. The resulting supernatant was discarded, and the pellet was saved at −75 °C.

### Analyses of lipid peroxidation

Lipid peroxidation was quantified in liver tissues and ER fraction lysates using a lipid hydroperoxide assay kit (Cayman Chemicals, Ann Arbor, MI, USA). In this assay, lipid hydroperoxide was extracted from the samples into chloroform using the extraction buffer provided by the manufacturer. The absorbance of each well was read at 500 nm using a 96 well plate spectrometer (SpectraMax 190, Molecular Devices, Sunnyvale, CA, USA). 13-Hydroperoxy-octadecadienoic acid was used as the standard. The cellular levels of lipid hydroperoxide were calculated as described by the manufacturer.

### Statistical analysis

Origin software (MicroCal, Northampton, MA, USA) was used for all statistical calculations. Results are presented as the mean ± SEM. Differences were tested for significance using one-way analysis of variance with Duncan’s multiple range test. Statistical significance was set at *P* < 0.05.

### Electronic supplementary material

Supplementary Information
