# Author Correction: Curcumin and *Curcuma longa* L. extract ameliorate lipid accumulation through the regulation of the endoplasmic reticulum redox and ER stress

**DOI:** 10.1038/s41598-024-55896-8

**Published:** 2024-03-22

**Authors:** Hwa-Young Lee, Seung-Wook Kim, Geum-Hwa Lee, Min-Kyung Choi, Han-Wool Chung, Yong-Chul Lee, Hyung-Ryong Kim, Ho Jeong Kwon, Han-Jung Chae

**Affiliations:** 1 Department of Pharmacology and New Drug Development Institute, Chonbuk National University Medical School, Jeonju, Chonbuk 561-180 Republic of Korea; 2 Chemical Genomics Global Research Laboratory, Department of Biotechnology, College of Life Science and Biotechnology, Yonsei University, Seoul, 120-752 Republic of Korea; 3 Department of Internal Medicine, School of Medicine, Chonbuk National University, Jeonju, 560-182 Republic of Korea; 4 Daegu Gyeonbuk Institute of Science and Technology (DGIST) Graduate School, Daegu, Gyeonbuk South Korea

Correction to: *Scientific Reports* 10.1038/s41598-017-06872-y, published online 26 July 2017

The original Article contains errors. Due to mistakes during Figure assembly, there are overlaps within two Figures and with previously published articles.

Within Figure 1e the panel “CL 300” is a duplication of panel “1d CCl4 + Cur”. In addition, the panel “4w Cur” overlaps with panel “Curcumin” of Figure 2 in Lee, HY et al.^1^.

**Figure 1:**
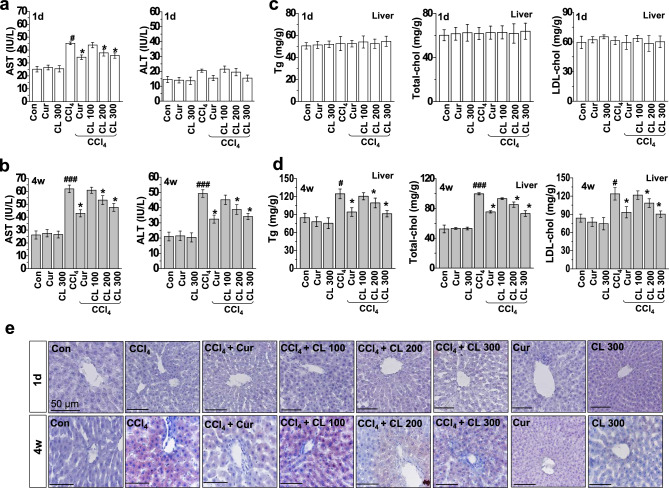
Curcumin and *Curcuma longa* L. extract regulate serum levels of AST and ALT and hepatic lipid accumulation in acute and chronic CCl_4_-models. Rats were intraperitoneally treated with CCl_4_ (0.1 mL/100 g, body weight) one time for (**a**) 1 day or (**b**) every other day for 4 weeks. Curcumin (200 mg/kg) or *Curcuma longa* L. extract (100, 200, or 300 mg/kg) was given each day for 3 days before CCl_4_ treatment and once daily after CCl_4_ treatment. Liver and blood samples were collected from all sacrificed animals. Six-h fasting serum levels of AST and ALT were determined. Six h fasting liver triglyceride, total cholesterol, and LDL-cholesterol levels were measured in the (**c**) 1 day and (**d**) 4 week CCl_4_-treated rats. (**e**) Representative images of liver sections from each group stained with hematoxylin–eosin and Oil-Red-O for lipid content. Scale bars = 50 µm. The experiments were repeated three times using tissues from at least three different rats. ^#^*P* < 0.05, ^###^*P* < 0.001 vs. the control group; *P < 0.01 vs. the CCl_4_ group (n = 10 rats per group). Cur: curcumin, CL: *Curcuma longa* L., AST: aspartate aminotransferase, ALT: alanine aminotransferase.

**Figure 2:**
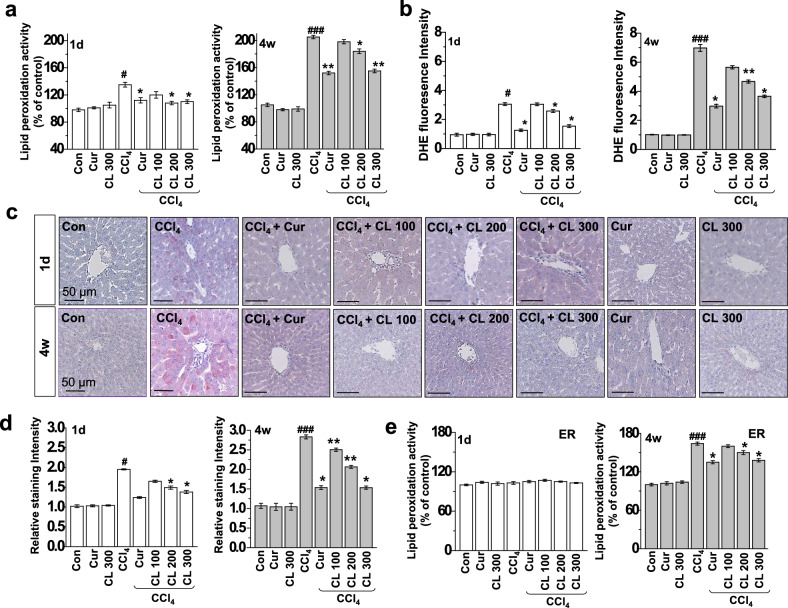
Curcumin and *Curcuma longa* L. extract regulate ROS accumulation in acute and chronic CCl_4_-models. Rats were intraperitoneally treated with CCl_4_ (0.1 mL/100 g body weight) one time for 1 day or every other day for 4 weeks. Curcumin (200 mg/kg) or *Curcuma longa* L. extract (100, 200, and 300 mg/kg) was given once daily. (**a**) Lipid peroxidation activity was measured in 1 day and 4 week CCl_4_-treated rats. (**b**) DHE staining in the liver was measured in 1 day and 4 week CCl_4_-treated rats. (**c**) Liver tissues from 1 day and 4 week CCl_4_-treated rats were stained with 4-HNE, and (**d**) the staining intensity of 4-HNE-positive cells was calculated. (**e**) Lipid peroxidation activity was measured in the ER fractions from the liver tissues of CCl_4_-treated rats. The experiments were repeated three times using tissues from at least three different rats. ^#^*P* < 0.05, ^###^*P* < 0.001 vs. the control group; *P < 0.01, **P < 0.05 vs. the CCl_4_ group (n = 10 rats per group). Cur: curcumin, CL: *Curcuma longa* L.

The corrected Figure 1 and accompanying legend appear below as Figure 1.

Within Figure 2c the panel “4w CL 300” duplicated panel “1d Con”, the panel “1d CCl4” is a duplication of “4w CCl4”, and the panel “4w CCL4 + CL 300” is duplicated from panel “1d CL 300”.

Additionally, in Figure 2c, the panel “4w Con” is similar to the panel “Control, Anti-4HNE” of Figure 4b in Lee, GH et al.^2^, the panel “4w CCl4 + CL 100” overlaps with “CCl4, Anti-4HNE” of Figure 4b in^2^, the panel “1d Cur” overlaps with “Curcumin + CCl4” of Figure 4b in^2^, and “4w Cur” shows similarities to “Curcumin, Anti-4HNE” of Figure 4b in^2^.

The corrected Figure 2 and accompanying legend appear below as Figure 2.
