# Supplementary material for: Curcumin and Curcuma longa L. extract ameliorate lipid accumulation through the regulation of the endoplasmic reticulum redox and ER stress
Source: Sci Rep. 2017 Jul 26;7:6513. doi: 10.1038/s41598-017-06872-y (PMC5529367; doi:10.1038/s41598-017-06872-y)
Supplement: Supplementary file 1 — Supplementary Information [file 41598_2017_6872_MOESM1_ESM.pdf]

Curcumin and *Curcuma longa* L. extract ameliorate lipid accumulation through the regulation of the endoplasmic reticulum redox and ER stress

Hwa-Young Lee[1$], Seung-Wook Kim[2$], Geum-Hwa Lee[1], Min-Kyung Choi[1], Han-Wool Chung[1], Yong-Chul Lee[3], Hyung-Ryong Kim[4], Ho Jeong Kwon[2*], Han-Jung Chae[1*]

[1]Department of Pharmacology and New Drug Development Institute, Chonbuk National University Medical School, Jeonju, Chonbuk 561-180, Republic of Korea; [2]Chemical Genomics Global Research Laboratory, Department of Biotechnology, College of Life Science and Biotechnology, Yonsei University, Seoul 120-752, Republic of Korea; [3]Department of Internal Medicine, School of Medicine, Chonbuk National University, Jeonju 560-182, Republic of Korea; [4]Department of Dental Pharmacology and Wonkwang Dental Research Institute, School of Dentistry, Wonkwang University, Iksan, Chonbuk, South Korea

*Correspondence to Ho Jeong Kwon, Chemical Genomics Global Research Laboratory, Department of Biotechnology, College of Life Science and Biotechnology, Yonsei University, Seoul 120-752, Republic of Korea, kwonhj@yonsei.ac.kr; Han-Jung Chae, Department of Pharmacology, New Drug Development Institute, Chonbuk National University, Medical School, Jeonju, Republic of Korea, Tel: 82-63-270-3092, Fax: 82-63-275-2855, E-mail: hjchae@jbnu.ac.kr

[$]These authors contributed equally to this work.

Supplementary Fig 1. Full scan mass spectrum of curcuminoids: (a) curcumin molecular weight (368.3), (b) bisdemethoxycurcumin (molecular weight 308.3), and (c) demethoxycurcumin (molecular weight 338.4). (d) LC-MS/MS chromatogram: *Curcuma longa* L. extract.

Supplementary Fig 2. Curcumin and *Curcuma longa* L. extract regulate hepatic injury in rats treated acutely or chronically with CCl_4. Rats were intraperitoneally treated with CCl_4 (0.1 mL/100 g body weight) one time for 1 day or every other day for 4 weeks. Curcumin (200 mg/kg) or *Curcuma longa* extract (100, 200, and 300 mg/kg) was given once daily. Representative images of liver sections from each group stained with (a) hematoxylin-eosin and (b) Sirius red collagen staining. Scale bars = 50 µm. The experiments were repeated three times using tissues from at least three different rats. $^{\#}P < 0.05$ vs. the CCl_4 group (n =10 rats per group). Cur: curcumin, CL: *Curcuma longa* L.*,* CV: central vein.

Supplementary Fig 3. Curcumin and *Curcuma longa* L. extract regulate lipid accumulation in rats treated acutely or chronically with CCl_4. Rats were intraperitoneally treated with CCl_4 (0.1 mL/100 g body weight) one time for (a-d) 1 day or (e-h) every other day for 4 weeks. Curcumin (200 mg/kg) or *Curcuma longa* L. extract (100, 200, and 300 mg/kg) was given once daily. (a, e) Six-hour fasting serum triglyceride, (b, f) total cholesterol, (c, g) LDL-cholesterol, and (d, h) HDL-cholesterol levels were measured in the 1 day and 4 week CCl_4-treated rats. The experiments were repeated three times using tissues from at least three different rats. $^{\#\#\#}P < 0.001$ vs. the control group; $^{*}P < 0.01$ vs. the CCl_4 group (n =10 rats per group). Cur: curcumin, CL: *Curcuma longa* L.

Supplementary Fig 4. Curcumin and *Curcuma longa* L. extract regulate hepatic lipid accumulation in rats treated acutely or chronically with CCl_4. Rats were intraperitoneally treated with CCl_4 (0.1 mL/100 g body weight) (a) one time for 1 day or (b) every other day for 4 weeks. Curcumin (200 mg/kg) or *Curcuma longa* L. extract (100, 200, and 300 mg/kg) was given once daily. Six-hour fasting liver HDL-cholesterol levels were measured in 1 day and 4 week CCl_4-treated rats. The experiments were repeated three times using tissues from at least three different rats. $^{\#}P < 0.05$ vs. the CCl_4 group (n =10 rats per group). Cur: curcumin, CL: *Curcuma longa* L., CV: central vein.

Supplementary Fig 5. Curcumin and *Curcuma longa* L. extract regulate antioxidant activity in rats treated acutely or chronically with CCl_4. Rats were intraperitoneally treated with CCl_4 (0.1 mL/100 g body weight) one time for 1 day or every other day for 4 weeks. Curcumin (200 mg/kg) or *Curcuma longa* L. extract (100, 200, or 300 mg/kg) was given once daily. After the livers were isolated, (a, b) GSH/GSSG ratios and (c, d) GPX levels were measured. The experiments were repeated three times using tissues from at least three different rats. $^{\#}P < 0.05$, $^{\#\#}P < 0.01$, $^{\#\#\#}P < 0.001$ vs. the control group; $^{*}P < 0.01$ vs. the CCl_4 group (n =10 rats per group). Cur: curcumin, CL: *Curcuma longa* L.

Supplementary Fig 6. Curcumin and *Curcuma longa* L. extract regulate antioxidant activity in rats treated acutely or chronically with CCl_4. Rats were intraperitoneally treated with CCl_4 (0.1 mL/100 g body weight) one time for 1 day or every other day for 4 weeks. Curcumin (200 mg/kg) or *Curcuma longa* L. extract (100, 200, and 300 mg/kg) was given once daily. (a, b) After the livers were isolated, SOD levels were measured. The experiments were repeated three times using tissues from at least three different rats. $^{\#}P < 0.05$ vs. the control group; $^{*}P < 0.01$ vs. the CCl_4 group (n =10 rats per group). Cur: curcumin, CL: *Curcuma longa* L.

Supplementary Fig 7. Curcumin and *Curcuma longa* L. extract regulate CCl_4-induced ER morphological alteration. Representative electron microcopy images with clear ER morphology. Cur: curcumin, CL: *Curcuma longa* L.

Supplementary Fig 8. CCl_4 induces lipid peroxidation and ER lipid peroxidation in primary hepatocytes. Mouse primary hepatocytes were exposed to 0.5% CCl_4 for 0, 1, 2, 4, 6, 12, or 24 h. (a) Lipid peroxidation analysis was performed in hepatocytes treated with CCl_4 for 0, 1, 2, 4, 6, 12 or 24 h. (b) Lipid peroxidation analysis was performed the ER fractions from hepatocytes treated with CCl_4 for 0, 1, 2, 4, 6, 12 or 24 h. $^{\#}P < 0.05$ vs. the control group. Cur: curcumin, CL: *Curcuma longa* L, 4-PBA: 4-Phenylbutyric acid, TUDCA: Tauroursodeoxycholic acid, NAC: N-Acetyl-L-cysteine.

Supplementary Fig 9. Curcumin and *Curcuma longa* L. extract have no effect on the ROS and ER stress response in normal hepatocytes. Mouse primary hepatocytes were exposed to 2 µM curcumin, 25 µM CL, 5 mM 4-PBA, 500 µg/mL TUDCA, or 2 mM NAC for 6 h or 12 h. Lipid peroxidation analysis (a) and DHE staining (b) were performed in the treated hepatocytes. (c) Immunoblotting was performed using antibodies against anti-GRP78, CHOP, p-eIF2α, eIF2α, p-PERK, PERK, p-JNK, JNK, p-IRE-1α, IRE1-α, and β-actin. $^{\#}P < 0.05$ vs. the control group. Cur: curcumin, CL: *Curcuma longa* L, 4-PBA: 4-Phenylbutyric acid, TUDCA: Tauroursodeoxycholic acid, NAC: N-Acetyl-L-cysteine.

Supplementary Figure 1

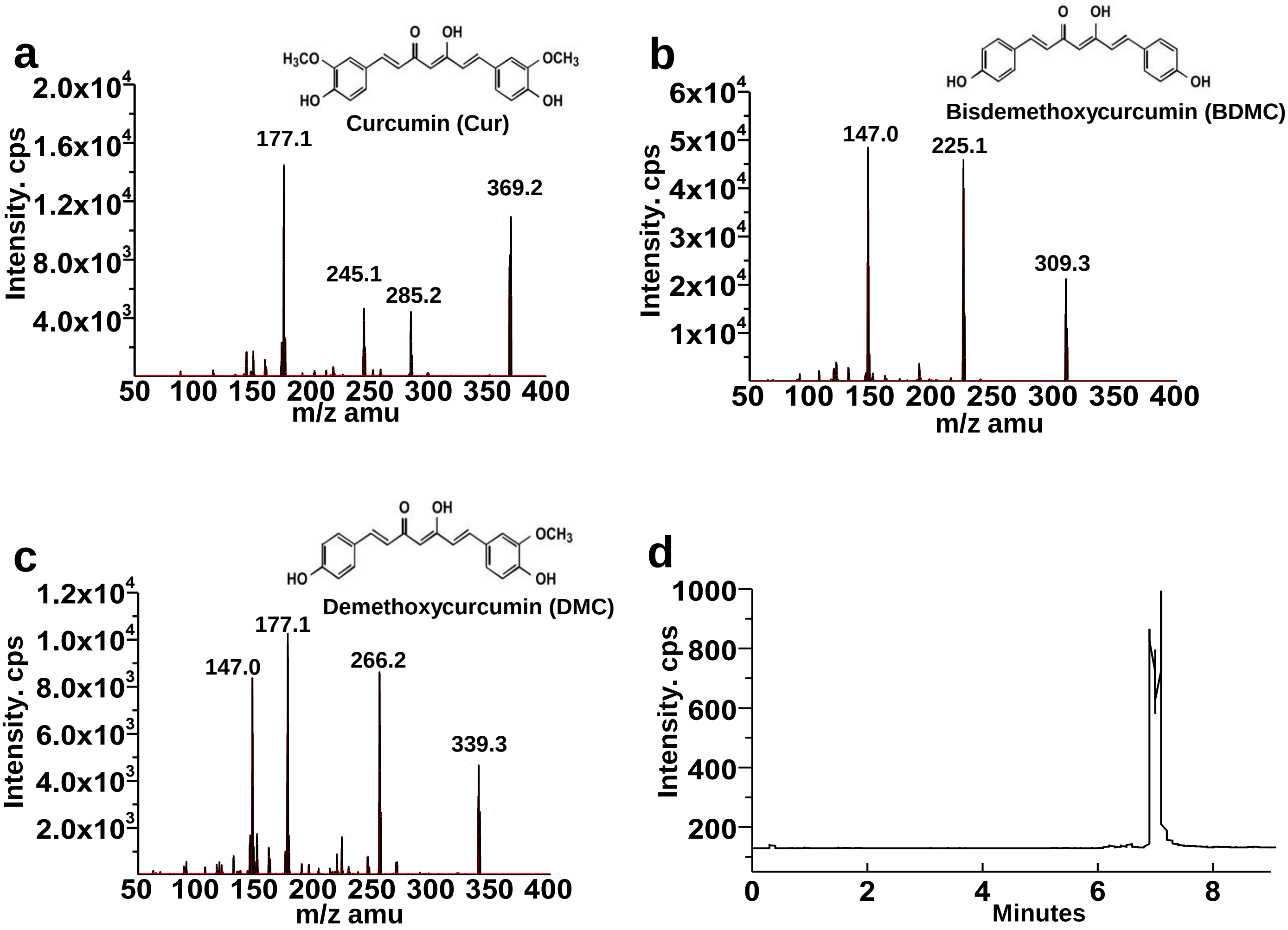

Supplementary Figure 2

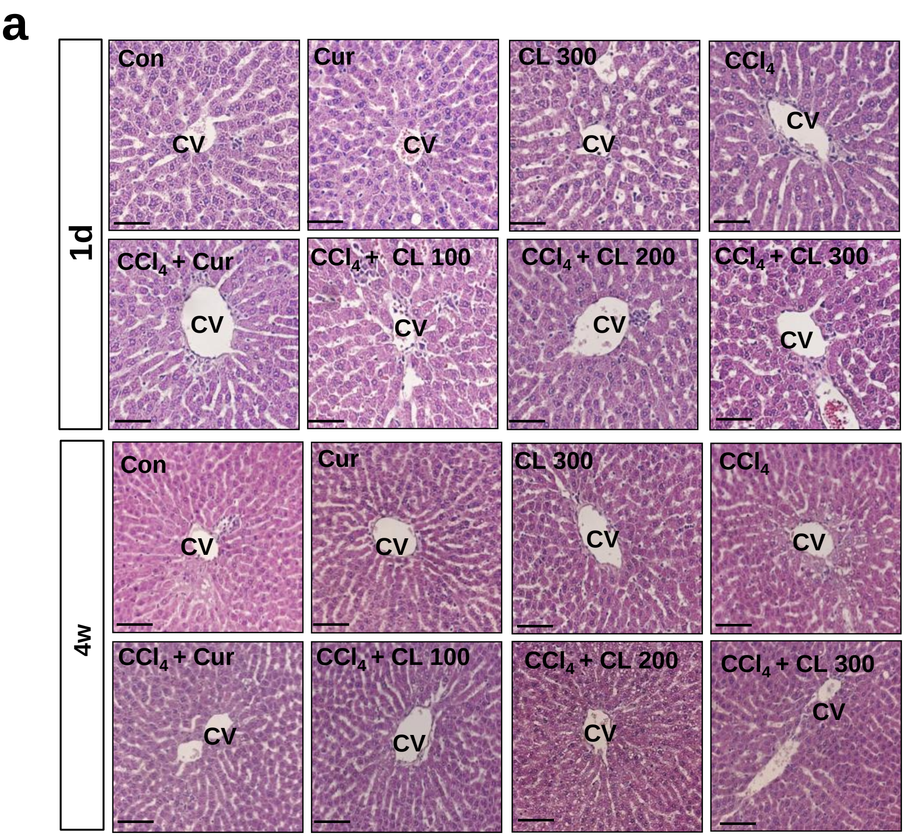

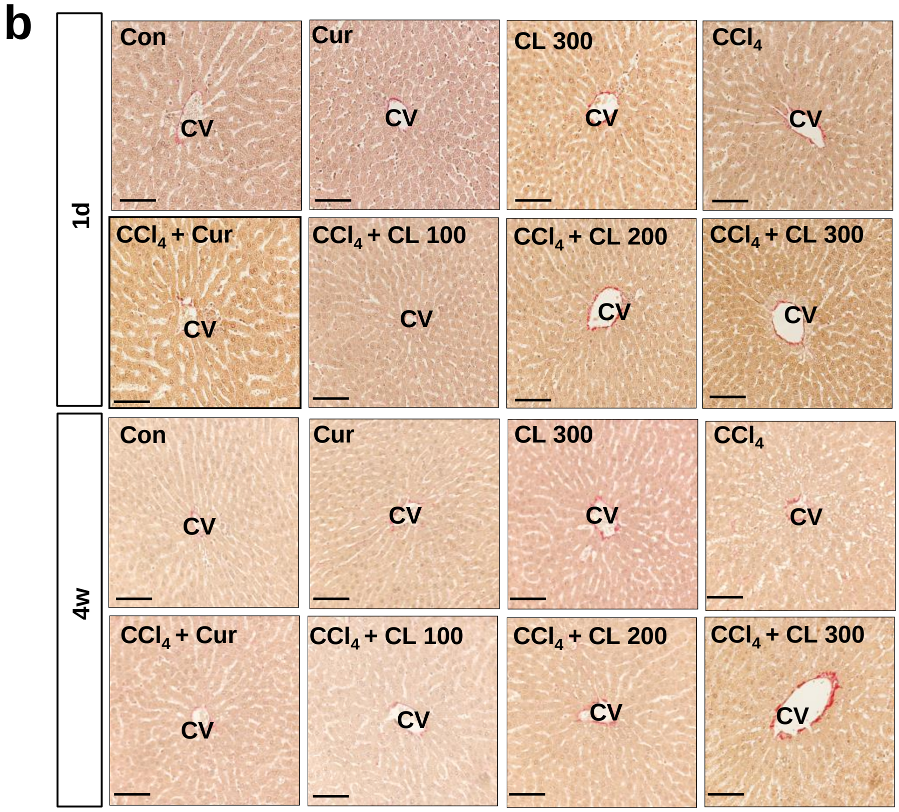

Supplementary Figure 3

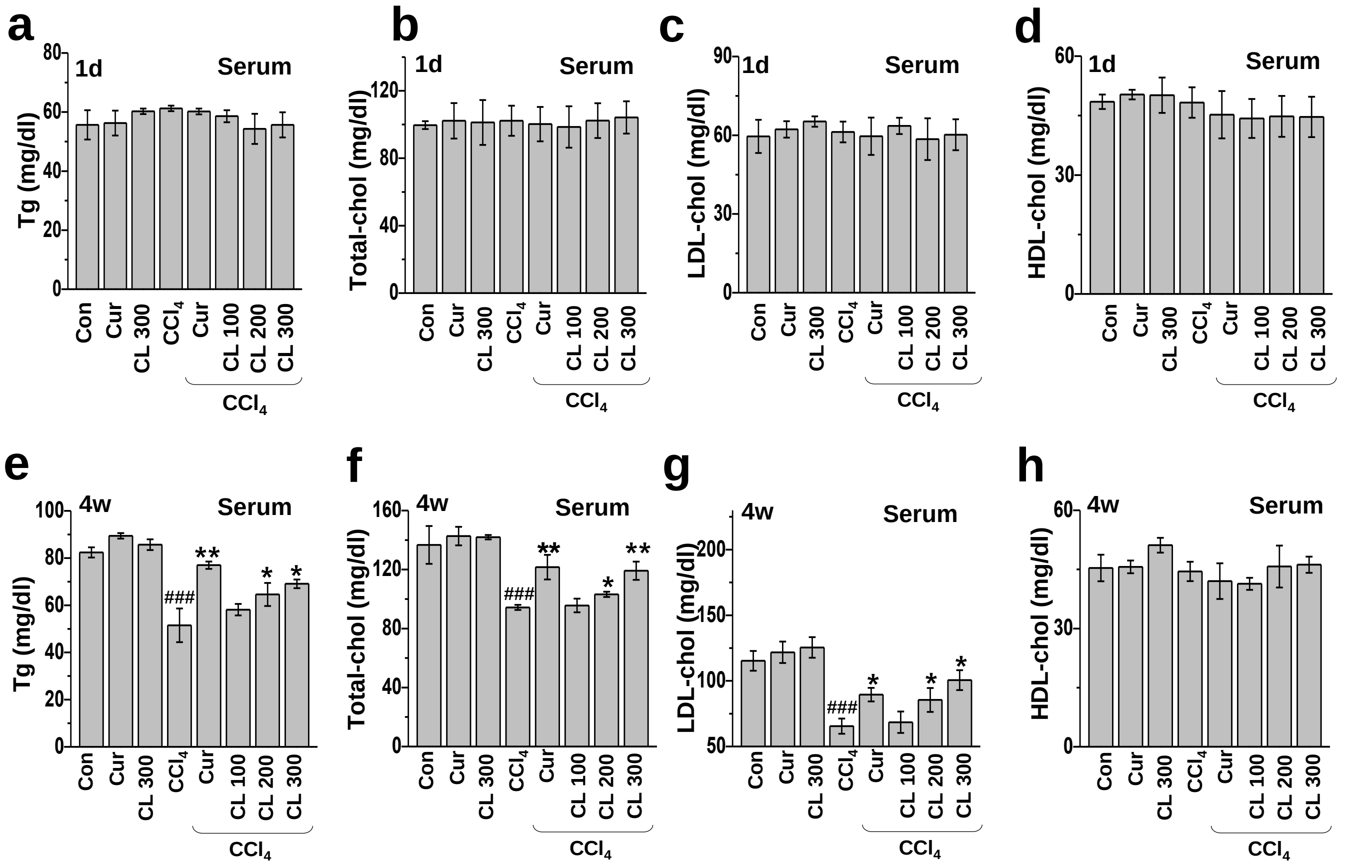

Supplementary Figure 4

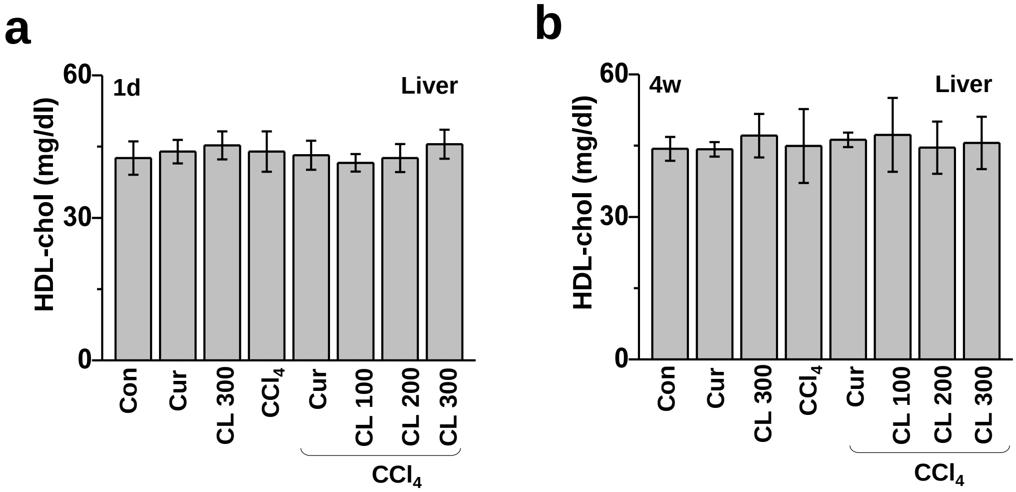

Supplementary Figure 5

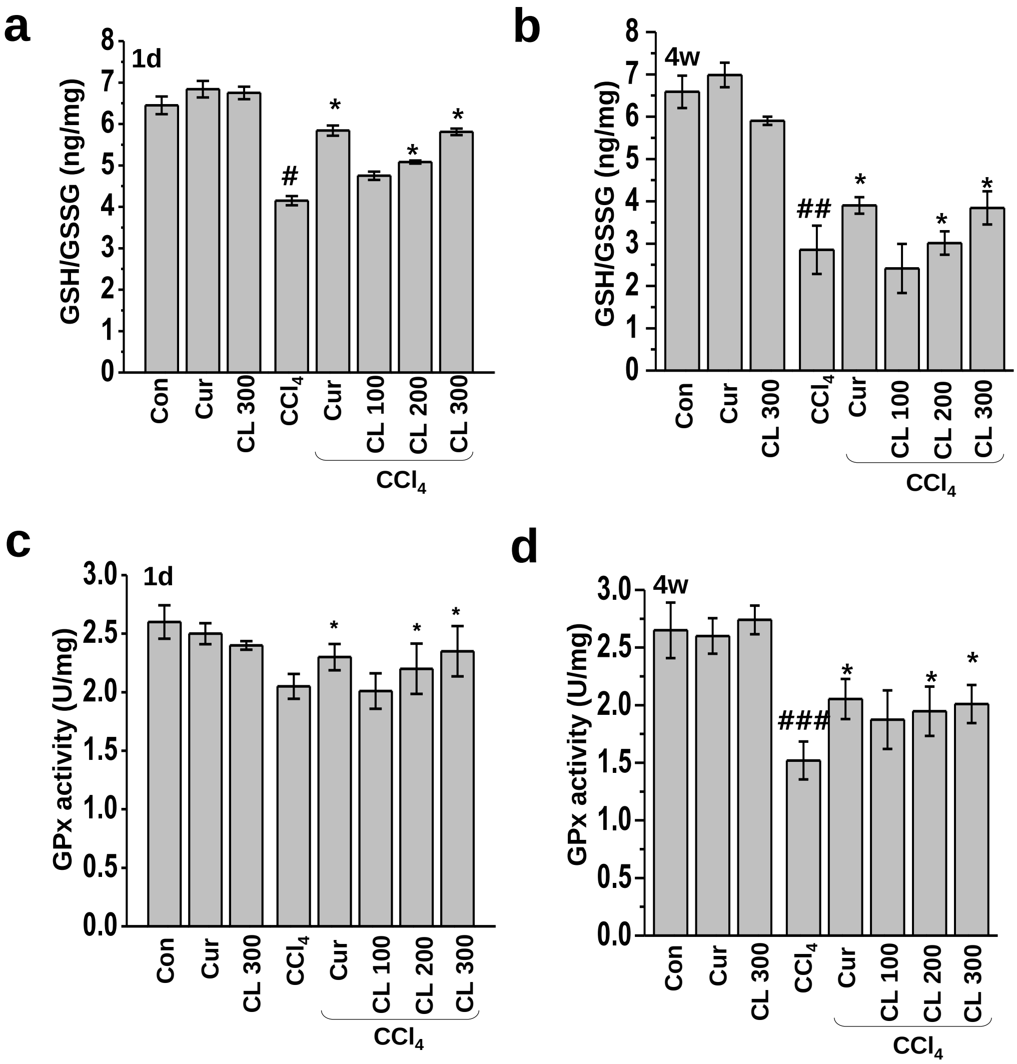

Supplementary Figure 6

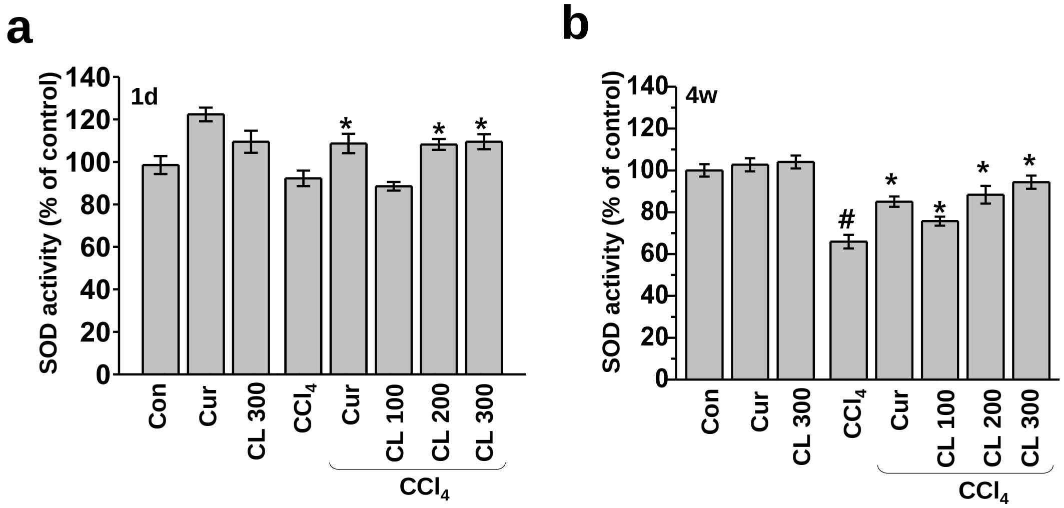

Supplementary Figure 7

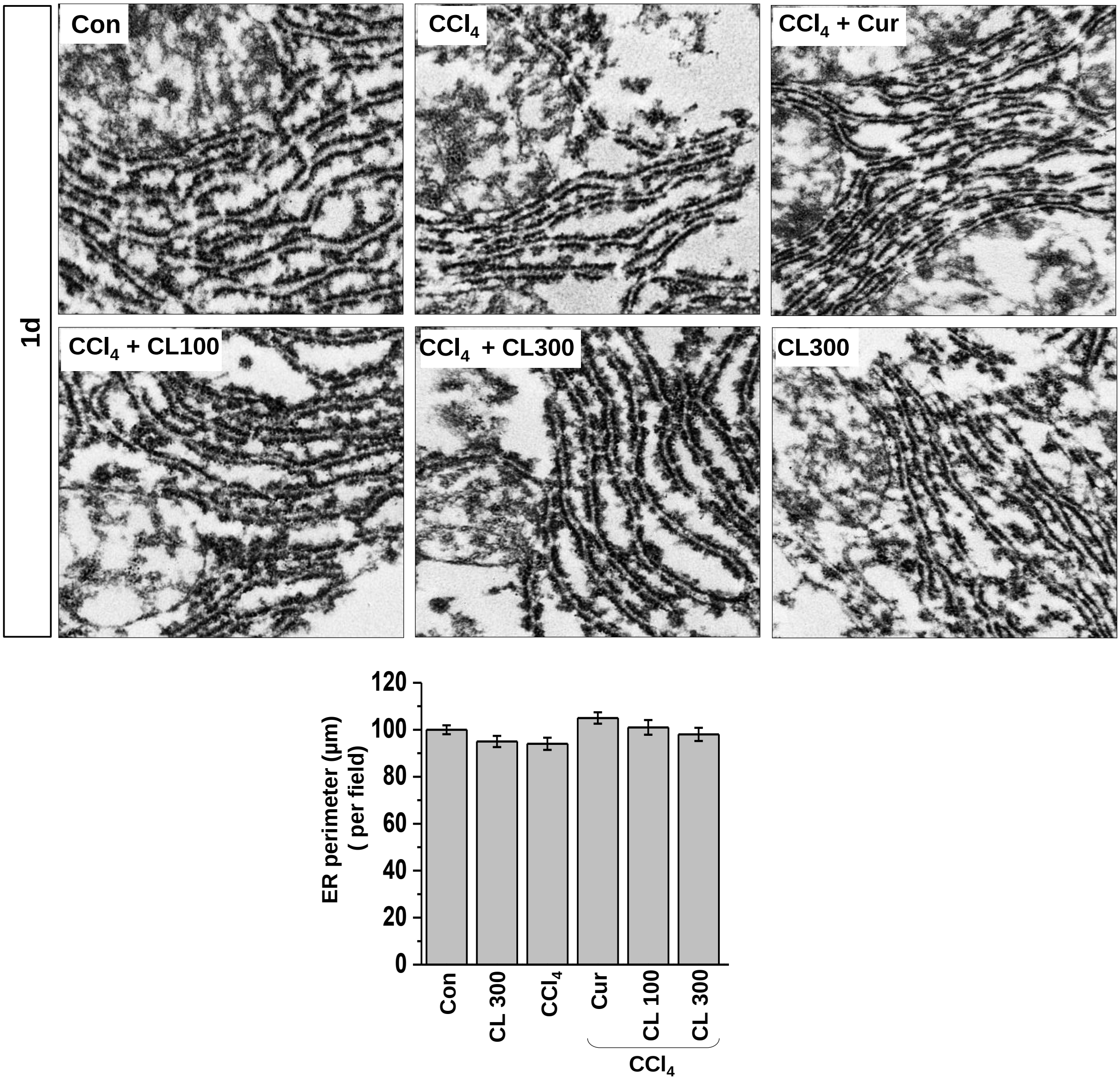

Supplementary Figure 8

a

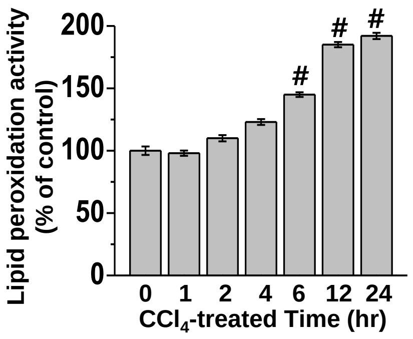

b

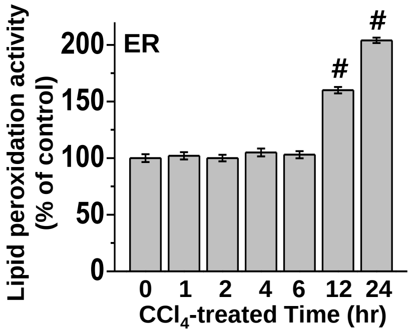

Supplementary Figure 9

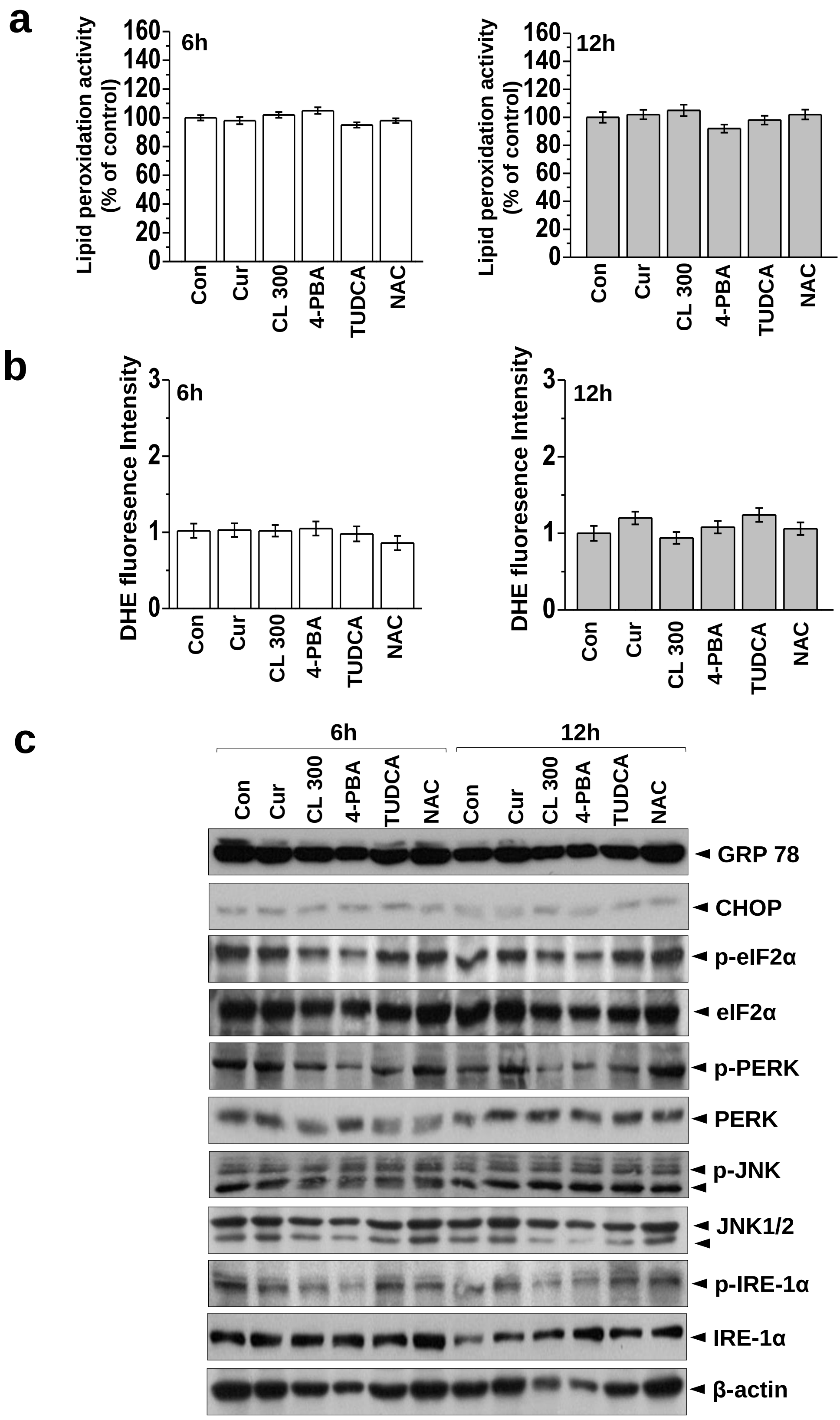

Supplementary Table 1 Analysis of *Curcuma longa* L. extract (India, Rajapuri)

Component	mg/100 g
**Bisdemethoxycurcumin**	1283.5 ± 8.5
**Demethoxycurcumin**	1284.6 ± 7.0
**Curcumin**	2269.2 ± 12.3
**Curcuminoids**	4837.2 ± 20.9

Supplementary Table 2 LC–MS/MS conditions for the acquisition in MRM ESI positive mode for the target compounds of *Curcuma longa* L. extract (India, Rajapuri).

Compound	Retention time (min)	Parent ion (m/z)	Transition ion (m/z)	Fragmentor Voltage (eV)	Collision Energy (eV)
**Bisdemethoxy curcumin**	7.0	309.1	225.1 [a]	110	10
			147.0 [b]	110	10
**Demethoxy curcumin**	7.3	339.1	266.2	110	10
			177.1	110	10
**Curcumin**	7.1	369.1	177.1	90	10
			285.2	90	10

a : Quantitation ion, b : Confirmation ion

Supplementary Table 3

Groups	1d			4w		
	Food Intake (g/day)	Body weight (g)		Food Intake (g/day)	Body weight (g)	
		Initial	Weight gain		Initial	Weight gain
Con	22.1±3.1	345.2±9.1	-0.2±8.6	42.3±2.2	328.2±20.3	39.3±9.6
Cur	23.2±3.3	344.3±12.6	0.7±9.5	45.8±1.8	330.5±15.9	21.8±14.2
CL 300	22.0±2.3	340.3±14.3	0.7±12.0	45.4±10.2	330.5±17.9	19.6±12.6
CCl_4	22.3±3.2	340.6±11.5	7.4±11.6	45.2±9.0	328.0±15.3	74.2±10.5#
CCl_4 + Cur	21.2±2.1	330.3±9.9	0.7±8.6	42.2±8.6	327.4±17.9	46.6±13.0
CCl_4 + CL 100	19.9±3.2	330.9±12.7	-0.9±5.7	47.1±9.1	327.1±18.6	71.8±12.0*
CCl_4 + CL 200	24.6±2.0	321.0±18.2	1.0±9.0	42.8±3.9	320.3±21.4	66.8±9.2*
CCl_4 + CL 300	23.1±1.3	331.3±14.6	2.7±13.3	43.0±8.7	331.4±16.3	45.2±8.6*

Con; control, Cur; curcumin, CL 100; *Curcuma longa* L. 100 mg/kg, CL 200; *Curcuma longa* L. 200 mg/kg, CL 300; *Curcuma longa* L. 300 mg/kg, #*P* < 0.05 vs. the control group; **P* < 0.01 vs. the CCl_4 group
